# Cost Analysis of the PARENT Trial of Community Health Workers in Early Childhood Preventive Care: A Secondary Analysis of a Cluster-Randomized Clinical Trial

**DOI:** 10.1001/jamanetworkopen.2025.22732

**Published:** 2025-07-31

**Authors:** Tumaini R. Coker, Sarah J. Lowry, Esha Dwibedi, Taylor Salaguinto, Peter G. Szilagyi, Kevin Fiscella, Sairan J. Rangel, Janette Ortiz, Marcia R. Weaver

**Affiliations:** 1 Seattle Children’s Research Institute, Seattle Children’s, Seattle, Washington; 2 Department of Pediatrics, University of Washington School of Medicine, Seattle; 3 Department of Health Metric Sciences, University of Washington, Seattle; 4 currently with Department of Public Health Sciences, University of Virginia, Charlottesville; 5 Department of Pediatrics, University of California, Los Angeles; 6 Department of Family Medicine, University of Rochester, Rochester, New York; 7 University of Washington School of Medicine, Seattle; 8 currently with Department of Family Medicine, Kaiser Permanente Los Angeles, Los Angeles, California; 9 currently with University of California, Davis, School of Medicine, Sacramento

## Abstract

**Importance:**

The Parent-Focused Redesign for Encounters, Newborns to Toddlers (PARENT) trial demonstrated increased anticipatory guidance and well child care visit (WCV) attendance but no effect on emergency department (ED) utilization.

**Objective:**

To assess whether the PARENT intervention was associated with lower non-WCV costs, and whether the increasing WCV attendance might offset recurring intervention costs.

**Design, Setting, and Participants:**

The PARENT cluster-randomized clinical trial was conducted from March 5, 2019, to July 14, 2022. This secondary cost analysis was conducted throughout the trial and continued to November 15, 2024. Participants included 937 enrolled parents with a child younger than 2 years presenting for a WCV at 10 clinics from 2 federally qualified health centers (FQHCs). Data on utilization and estimated cost of care using a unit cost approach were collected. Parents were interviewed at baseline and 6 and 12 months and asked about the number of encounters and the child’s diagnosis for subspecialty referrals, urgent care visits, ED visits, and hospitalizations.

**Intervention:**

PARENT is a team-based approach to health care that uses a trained community health worker as part of the well child care team to provide preventive care services to children aged 0 to 2 years.

**Main Outcomes and Measures:**

The main outcome of this analysis was 2016 unit cost estimates for subspecialty referrals, urgent care visits, ED visits, and hospitalizations by health condition and child’s age using data from the US Disease Expenditure Study.

**Results:**

Among 937 enrolled PARENT participants, 785 (83.8%) completed the 12-month interview and were included in analyses. Mean (SD) child age at enrollment was 4.4 (4.0) months, and most participants were mothers (868 of 914 with available data [95.4%]) and were Medicaid insured (855 of 914 with available data [93.5%]). The intervention group had statistically significant lower costs than the control group for both subspecialty referrals (−$213; 95% uncertainty interval [UI], −$540 to −$106) and total cost of urgent care visits, ED visits, and hospitalizations (−$70; 95% UI, −$150 to −$13). Based on the mean (SD) volume of newborns across all participating clinics (5.8 [4.1] newborns per week), marginal revenue from increased WCV attendance was greater than annual community health worker salary and benefits costs under select current reimbursements.

**Conclusions and Relevance:**

This secondary analysis of a cluster-randomized clinical trial suggests that the cost of the PARENT intervention was offset by savings in non-WCV health care utilization, and revenue for federally qualified health centers from increased WCV attendance could fund ongoing intervention costs.

**Trial Registration:**

ClinicalTrials.gov Identifier: NCT03797898

## Key Points

**Question:**

The Parent-Focused Redesign for Encounters, Newborns to Toddlers (PARENT) trial intervention increased well child care visits, but was the increase associated with lower overall costs of health care, and would increasing visit attendance offset intervention costs?

**Findings:**

In this secondary analysis of data from 785 participants of the PARENT cluster-randomized clinical trial, health care (urgent care, emergency department visits, hospitalizations, and subspecialty referrals) costs were lower for intervention vs control groups.

**Meaning:**

These findings suggest that the PARENT intervention decreased the cost of other health care services, and within federally qualified health centers, additional revenue due to the increased well child care visit adherence may help to fund the intervention costs.

## Teaser

This secondary analysis of a cluster-randomized clinical trial assesses costs for subspecialty referrals, urgent care visits, emergency department visits, and hospitalization among children younger than 2 years following an intervention incorporating a community health worker as part of the well child care team.

## Introduction

The Parent-Focused Redesign for Encounters, Newborns to Toddlers (PARENT) intervention is a team-based approach to well child care that adds a trained community health worker (CHW) to the well child care visit (WCV) team to provide comprehensive family-centered WCV services, address concerns related to family social needs, and decrease reliance on the clinician as the sole provider of preventive care services.^1,2,3^ A randomized clinical trial (RCT) of the PARENT approach at 2 pediatric practices serving a low-income, predominately Latino population demonstrated robust improvements in the receipt of preventive care services (eg, social needs, developmental screening, and health education and guidance), experiences of care (eg, family-centeredness and helpfulness of care), and reduced emergency department (ED) visits.^2^

In a larger, cluster RCT, investigators estimated the effectiveness of the PARENT intervention among 10 clinics from 2 federally qualified health centers (FQHCs) in California and Washington randomly assigned to the PARENT intervention or control.^4^ The PARENT intervention improved the receipt of preventive care services and increased WCV attendance.^4^ The RCT did not find an intervention effect on ED utilization.

For the present study, our first objective was to conduct a cost analysis of the PARENT trial, focused on health care utilization and costs outside WCVs. Our a priori hypothesis was that the costs of the intervention would be offset by lower total costs due to decreased ED utilization^5^; however, because of the null ED utilization findings,^4^ we also examined a post hoc hypothesis that intervention participants have lower costs for all non-WCV health care utilization. Due to the finding that WCV attendance increased,^4^ we also examined a post hoc hypothesis that increased WCV revenue could offset recurring intervention costs for FQHCs.

## Methods

### Cost Analysis

The cluster RCT was conducted between March 5, 2019, and July 14, 2022, with 10 clinical sites of 2 large FQHCs randomized to control or intervention. Initial trial findings and those of the present analysis were reported in concordance with the Consolidated Standards of Reporting Trials (CONSORT) guideline (eFigure in Supplement 1); the trial protocol is found in Supplement 2 and was published previously.^5^ Cost data were collected throughout the trial, and the analysis, conducted from the health system perspective, continued to November 15, 2024. The study was approved by the Seattle Children’s Institutional Review Board. Informed consent was obtained in the original trial either in person or over the telephone through a research assistant.

Parents or caregivers (henceforth termed *parents*) were enrolled when their child was 12 months or younger and presenting for a WCV. They were interviewed at baseline and 6 and 12 months and asked about the number of and reasons for health care encounters. We focused on subspecialty referrals received from primary care, urgent and acute (henceforth termed *urgent*) care visits, ED visits, and hospitalizations. Unit cost estimates for 6 health services by health condition and age are available as the US Disease Expenditure Study (DEX)^6^ estimate of 2016 spending divided by the corresponding number of 2016 encounters. Based on parent-reported data, each encounter was mapped to 1 of the 154 health conditions with DEX estimates and assigned a unit cost. Total spending at baseline and during the child’s 12-month period in the trial was the sum of unit costs across encounters. In regression analysis, we used individual data on each child’s total cost to test the difference in cost between intervention and control.

### Data Collection

At each interview (baseline and 6 and 12 months post enrollment), parents were asked about their child’s use of health care during a specified time period.^5^ The time period was 3 months for the baseline interview and 6 months each for the 6- and 12-month interviews. If a child was younger than 3 months at baseline, parents were asked about postdischarge health care utilization after birth. If the parent did not complete the 6-month interview, they were asked at 12 months about the full 12 months after baseline. Parents reported utilization for each service type, including number of encounters and details for each (location, date, and reason) (eTable 1 in Supplement 1).

### Coding Encounters With DEX Data

US DEX provides the only estimates of cost per encounter by service, condition, age, and year for the US.^6^ Three authors (T.R.C., T.S., and S.J.R.) matched all ED encounters with 1 of 154 DEX health conditions. We initially coded the 6-month ED encounters, creating a codebook that matched each diagnosis (based on parent-reported encounter information) to a DEX health condition and included a definition for each code. Authors made adjustments to the codebook to ensure that parent-reported diagnoses (eg, fever with diarrhea) were as closely matched to a DEX health condition (eg, diarrheal diseases or intestinal infectious disease) as possible. We then iteratively coded the 6-month responses for urgent care, subspecialty referrals, and hospitalizations using the codebook, editing definitions, and DEX matches. Following feedback from additional authors, the baseline and 12-month follow-up data were coded using the codebook.

DEX results are reported at 3 levels of aggregation. We matched each parent-reported diagnosis to the most detailed condition, such as otitis media. If the parent-reported diagnosis, such as hand-foot-and-mouth disease, did not match a DEX condition at this level, we looked for a match at the next highest level of aggregation, such as communicable, maternal, neonatal, and nutritional disorders. The most aggregated condition is all conditions, for which the mean is calculated across all conditions. This process was guided by codes from the *International Classification of Diseases, Ninth Revision* (*ICD-9*), and *International Statistical Classification of Diseases and Related Health Problems, Tenth Revision* (*ICD-10*), corresponding to the parent-reported diagnosis, and the DEX map of *ICD-9* and *ICD-10* codes to conditions.

### Unit Cost Calculation

Unit costs were the cost per unit of service; we referred to a unit of service as an encounter. Unit costs were calculated as the DEX estimate of 2016 spending by service, health condition, and age divided by the corresponding number of 2016 encounters. DEX estimates from 1996 to 2016 are based on 6 nationally representative sources, including the Medical Expenditure Panel Survey data, Health MarketScan Commercial Claims and Encounters data, and 7 additional sources.^6^ Estimates of health care spending and encounters are reconciled across multiple sources, and all estimates are scaled to National Health Expenditure Account totals.^7^ In the DEX estimates, health care spending across 21 years was converted to 2016 US dollars using the Gross National Product Price Index. They can be converted to more recent years by multiplying each result by a fixed proportion, based on the price index for that year. For example, 2016 results can be converted to 2024 US dollars by multiplying each result by 1.198.^8^

A DEX unit cost was assigned to each encounter based on DEX condition and age for each of the 4 services: urgent care visits, ED visits, hospitalizations, and subspecialty visits. We used the same DEX ambulatory care unit cost estimates for urgent care and subspecialty visits. Since DEX estimates also vary based on child age, we matched age-specific estimates for each encounter with the child’s age on the date of the interview. The sum across subspecialty encounters gives subspecialty costs for each child. Similarly, the sum across encounters for urgent care, ED visits, and hospitalizations gives the total cost of other services for each child. For baseline data, we calculated each child’s cost per week, because the time between birth and baseline varied across children. We combined 6- and 12-month data to calculate cost estimates for the 12-month period in the trial.

### Uncertainty in DEX Estimates

DEX conveys the strength of the evidence from multiple sources by taking 1000 draws from the posterior distribution of each estimate to create an uncertainty distribution for each result and report a 95% uncertainty interval (UI). A draw is a number randomly selected from the distribution, where a number that occurs more frequently is more likely to be selected. To propagate uncertainty in our analysis, 1000 draws for each of the unit costs were calculated with DEX results for 1000 draws for spending and 1000 draws for number of encounters; for example, the first draw of the unit cost was the ratio of the first draw for spending divided by the first draw for encounters. eTable 2 in Supplement 1 shows the frequency, mean unit cost, SD, and coefficient of variation of the 10 DEX conditions that were most frequently reported by parents, by service type and age category.

### FQHC Revenue Model

In the PARENT trial, intervention participants were more likely to have completed all WCVs in a 12-month period compared with control participants.^5^ In the Prospective Payment System (PPS)^9,10,11^ for FQHCs, each WCV is reimbursed at a specified rate. Consequently, increased WCV attendance translates directly to additional revenue. We obtained the WCV 2021 PPS rate for each clinic and calculated additional revenue from greater WCV attendance, based on national guidelines for WCV periodicity,^12^ by age and a cohort of children who entered at birth and progressed through visits at each age. Our estimates used the intervention WCV attendance rates, the observed cohort size (minimum, mean, and maximum), and the 4 levels of payment (PPS) rates for the trial’s 10 participating clinics.

To estimate recurring intervention costs, we used CHW salary and benefits at each of the clinics. Each CHW tracked their time devoted to intervention activities (including non–patient facing time such as follow-up and record keeping) on a random sample of days. These activities did not include training time, because this is a fixed cost, and the decision about continuing a service is based on variable costs. The recurring cost of the CHW was multiplied by the percentage of time devoted to the intervention and compared with the additional revenue.

### Statistical Analysis

Health care costs were not normally distributed, because a substantial proportion of the sample may have zero costs, and costs are right-skewed among people who incur them. To account for these characteristics, 2-part model estimates were performed, where the first part estimated the probability of having encounters and consequently costs, and the second part was a conditional model of the log-transformed costs among patients with encounters.^13,14,15,16^ We conducted a White test for heteroscedasticity and rejected the hypothesis that the residuals were heteroscedastic in any of the independent variables.^17^ Consequently, we used the Duan smearing estimator for retransformation.^13,15^ The combined estimates were the product of the estimated probability and cost.

Our model covariates were intervention and control arms, baseline spending per week, and child’s age at enrollment. At baseline, child age and mean cost per week were significantly different between the intervention and control arms. Regarding missing outcomes data, we compared baseline characteristics of participants with 12-month survey data and those missing 12-month data.^4^ We conducted a complete case analysis of the children with 12-month survey data. This approach assumed data were missing at random. However, if data were not missing at random, then results could be biased.^18^

We conducted 2 sensitivity analyses. First, we excluded 1 outlier child with much higher costs at baseline due to cardiac surgical procedures, and much higher subspecialty referral costs during the trial due to cardiovascular disease visits. Second, we winsorized the outliers by replacing the lowest 5% of costs across all draws with the value at the fifth percentile and the highest 5% with the value at the 95th percentile.^19^

The 2-part model estimates were performed for each of the 1000 samples. For each sample, the model estimated a coefficient with an SE for parts 1 and 2 and estimated the difference in the combined total cost of intervention vs control with a 95% CI. These statistics were for the intervention effect if the unit cost was known with certainty. The main results for parts 1 and 2 and the combined total cost were from the 1000 estimates. We reported the mean and a 95% UI; where the estimates from each sample were ranked from smallest to largest, the 95% UI represented the 25th and 97.5th estimate. The bootstrap UI reflects the uncertainty in the coefficient and the uncertainty in the data on health care costs. The bootstrap UI was roughly twice as wide as the CI. If the bootstrap UI showed a statistically significant difference between the intervention and control groups, results for each of the 1000 samples were also statistically significant, and the latter were not shown.

## Results

Of 937 parents enrolled in the trial, 785 (83.8%) completed the 12-month interview and were included in our analysis; retention rates were similar for study arms. A total of 378 participants were included in the intervention group and 407 in the control group. Participant characteristics are reported in Coker et al.^1^ In brief, mean (SD) child age at enrollment was 4.4 (4.0) months, and 855 of 914 participants with available data (93.5%) were Medicaid insured. A total of 868 enrollees (95.4%) were mothers, 42 (4.6%) were fathers, and 1 (0.2%) were grandmothers.

### Descriptive Statistics

At baseline, the proportion of children with any subspecialty encounters was lower in the intervention than control groups (15 [4.0%] vs 19 [4.7%]), and the proportion with at least 1 urgent care visit, ED visit, or hospitalization was also lower (78 [20.6%] vs 97 [23.8%]) (Table 1), consistent with the younger age of intervention participants (mean [SD], 4.0 [4.0] vs 4.7 [4.0] months). The mean (SD) baseline cost per week was higher in the intervention than control group for subspecialty visits ($61.5 [$1363.1] vs $40.7 [$737.4]), and for total cost of urgent care, ED, and hospitalizations ($536.1 [$25 939.9] vs $212.2 [$1753.5]). The SD of the cost estimates at baseline was high, especially for the intervention group, where the mean subspecialty referral cost for 1 participant was 4 times higher than the next highest cost.

**Table 1.** Descriptive Statistics on Services and Cost Utilization at Baseline and 12 Months^a^

Statistic	Study arm		
	Intervention	Control	All
No. enrolled	452	485	937
Excluded from sample, No. (%)	74 (16.4)	78 (16.1)	152 (16.2)
No. completed 12-mo interview	378	407	785
**Subspecialty visits**			
Baseline			
Nonzero cost, No. (%)	15 (4.0)	19 (4.7)	34 (4.3)
Cost per week in past 3 mo or since birth, $ US			
Mean (SD)	61.5 (1363.1)	40.7 (737.4)	50.7 (1084.8)
Median (IQR)	0.0 (0.0-0.0)	0.0 (0.0-0.0)	0.0 (0.0-0.0)
12-mo intervention period			
Nonzero cost, No. (%)	59 (15.6)	59 (14.5)	118 (15.0)
Total costs during 12 mo, $ US			
Mean (SD)	875.4 (12 377.1)	704.5 (3942.4)	786.8 (9046.1)
Median (IQR)	0.0 (0.0-0.0)	0.0 (0.0-0.0)	0.0 (0.0-0.0)
**Urgent care visits, ED visits, and hospitalizations**			
Baseline			
Nonzero cost, No. (%)	78 (20.6)	97 (23.8)	175 (22.3)
Cost per week in past 3 mo or since birth, $ US			
Mean (SD)	536.1 (25 939.9)	212.2 (1753.5)	368.2 (18 045.2)
Median (IQR)	0.0 (0.0-0.0)	0.0 (0.0-0.0)	0.0 (0.0-0.0)
12-mo intervention period			
Nonzero cost, No. (%)	205 (54.2)	230 (56.5)	435 (55.4)
Total costs during 12 mo, $ US			
Mean (SD)	1683.8 (23 630.7)	2246.6 (18 151.2)	1975.6 (20 971.1)
Median (IQR)	420.4 (0.0-1231.0)	524.0 (0.0-1426.2)	489.1 (0.0-1343.3)

Abbreviation: ED, emergency department.

^a^ Results are in 2016 US dollars. To convert to 2024 US dollars, multiply each result by 1.198.^7^

During the 12-month trial period, 59 intervention participants (15.6%) and 59 control participants (14.5%) had costs for subspecialty visits, with the mean (SD) subspecialty costs during the 12-month period at $875.4 ($12 377.1) for intervention and $704.5 ($3942.4) for control participants. During this same period, 205 intervention participants (54.2%) and 230 control participants (56.5%) had costs for urgent care, ED, or hospitalization. The mean (SD) costs during the 12 months for intervention was $1683.8 ($23 630.7) and for control participants was $2246.6 ($18 151.2) (Table 1).

### Two-Part Model

After controlling for baseline age and subspecialty costs in part 1 of the analysis (Table 2), the intervention group had statistically significant higher odds of at least 1 (compared with none) subspecialty referral compared with the control group (odds ratio [OR], 1.086; 95% UI, 1.078-1.092) during the trial. In part 2, among participants with subspecialty referrals, the intervention decreased the total cost of subspecialty visits (coefficient, −0.350; 95% UI, −0.420 to −0.283). The net effect of these results was that subspecialty combined total cost was lower for the intervention than control groups (Table 2). The mean difference between the intervention and control groups was statistically significant (−$213; 95% UI, −$540 to −$106) and would total $80 514 (95% UI, $40 068-$204 120) for the 378 intervention group participants (Table 2).

**Table 2.** Two-Part Model Results^a^

Analysis	Effect of intervention		Combined total cost, mean, US $					Estimated savings per year, $ US (95% UI)^c^
	Probability of services during 12 mo, OR (95% UI)	Total cost among children with services, coefficient (95% UI)	Intervention	Control		Mean difference (95% UI)^b^		
**Subspecialty visits**								
Main analysis	1.086 (1.078 to 1.092)	−0.350 (−0.420 to −0.283)	638		850		−213 (−540 to −106)	80 514 (40 068 to 204 120)
Sensitivity analysis A^d^	1.069 (1.062 to 1.076)	−0.446 (−0.539 to −0.370)	462		687		−26 (−485 to −127)	85 428 (48 006 to 183 330)
Sensitivity analysis B^e^	1.086 (1.078 to 1.092)	−0.379 (−0.451 to −0.313)	431		589		−158 (−221 to −107)	59 724 (40 446 to 83 538)
**Urgent care visits, ED visits, and hospitalizations**								
Main analysis	0.926 (0.916 to 0.935)	−0.004 (−0.037 to 0.024)	1867		1937		−70 (−150 to −13)	26 460 (4914 to 56 700)
Sensitivity analysis A^d^	0.919 (0.909 to 0.929)	−0.005 (−0.049 to 0.038)	1867		1943		−76 (−156 to −18)	28 728 (6804 to 58 968)
Sensitivity analysis B^e^	0.926 (0.916 to 0.935)	−0.004 (−0.030 to 0.021)	1053		1093		−40 (−70 to −12)	15 120 (4536 to 26 460)

Abbreviations: ED, emergency department; OR, odds ratio; UI, uncertainty interval.

^a^ Results are in 2016 US dollars. To convert them to 2024 US dollars, multiply each result by 1.198.^7^

^b^ The difference between groups and the total mean difference result may not be the same due to rounding.

^c^ Calculated as the mean difference in total cost multiplied by 378, which is the number of participants in the intervention group.

^d^ Excluded 1 patient with much higher costs at baseline, due to cardiac surgical procedures. This changes the coefficient estimates for the entire sample, which causes the estimated costs and probabilities in the combined effect to be different for intervention and control groups.

^e^ The top 5% of costs across all draws was replaced with the 95th percentile of cost of all draws and the bottom 5% of costs was replaced with the 5th percentile of costs.

In bivariate descriptive analysis, intervention participants had higher subspecialty costs, whereas in the multivariate analysis, intervention participants had lower costs when compared with control participants. This was due in part to the log transformation of the cost variable in the 2-part model. The transformation gives less weight to 1 participant whose subspecialty costs for cardiovascular disease were an outlier. The range of unit cost estimate for cardiovascular disease was wider than for other conditions; the coefficient of variation was 1.20, whereas most were less than 0.20 (eTable 2 in Supplement 1). In sensitivity analysis A, in which this participant was removed from the analysis, and sensitivity analysis B with winsorized outliers, the conclusions were similar, but difference in total costs was larger in the former and smaller in the latter (Table 2).

In the 2-part model estimates for the total cost of urgent care, ED, and hospitalizations, the intervention group had statistically significant lower odds of at least 1 encounter compared with the control group (OR, 0.926; 95% UI, 0.916-0.935). In part 2, among participants with encounters, the cost was lower in the intervention than control groups (coefficient, −0.004; 95% UI, −0.037 to 0.024), but the difference was not statistically significant. The net effect on the combined total cost of other care was that the mean difference between the intervention and control groups was statistically significant (−$70; 95% UI, −$150 to −$13) (Table 2) and would total $26 460 (95% UI, $4914-$56 700) for 378 participants. In sensitivity analysis A, in which the same participant was removed from the analysis, and sensitivity analysis B with winsorized outliers, the conclusions were similar, but difference in total costs was larger in the former and smaller in the latter. (Table 2).

### Revenue Model Results

FQHC records provided full-time salary and benefits for the CHWs in California ($67 505) and Washington ($62 400) clinics during the first year of the trial. Well child care per-visit payment rates varied by clinic site for the California FQHC ($163 for 1 site, $199 for 3 sites, and $221 for 2 clinic sites). The 4 Washington clinics received 1 per-visit rate ($376). The benefit (in per-visit payment) from the intervention effect on WCV attendance increased as the size of the cohort presenting for WCVs at the clinic increased (Table 3). Revenue from additional WCVs due to higher attendance was calculated for different payment rates and 3 cohort sizes that represent the total number of newborn visits each week in each clinic observed during the trial; lowest was 2.7 and highest was 13.5, and the mean (SD) across all participating clinics was 5.8 (4.1) newborns per week.

**Table 3.** Revenue From Per-Visit Encounter Payment Attributed to Intervention Impact on Increasing WCV Attendance

Clinic volume (No. of newborn visits/wk)	Additional WCV in intervention^a^	Per-visit encounter payment attributed to intervention, $ US			
		California			Washington, $376 PPS per WCV (4 clinics)
		$163 PPS per WCV (1 clinic)	$199 PPS per WCV (3 clinics)	$221 PPS per WCV (2 clinics)	
Minimum (2.7)	105	17 002	20 814	23 084	39 288
Mean (5.8)	224	36 443	44 601	49 465	84 189^b^
Maximum (13.5)	522	85 011^b^	104 069^b^	115 418^b^	196 440^b^

Abbreviations: PPS, Prospective Payment System; WCV, well child care visit.

^a^ Represents the number of additional WCVs, calculated based on the difference in WCV attendance between intervention and control groups observed in the randomized clinical trial.^4^

^b^ Indicates the revenue from additional WCVs would exceed the costs of the community health worker (CHW; using full-time salary and benefits for the CHWs in California [$67 505] and Washington clinics [$62 400]) for newborn volume and clinic site.

Calculations with the mean cohort size across clinics (5.8 newborns/week) would sustain the PARENT coach with the Washington reimbursement rate for WCVs (Table 3). The highest observed cohort size (13.5 newborns/week) would sustain the PARENT coach across all clinics, including those with the lowest reimbursement rate for WCVs.

## Discussion

In this secondary analysis of a cluster RCT, we analyzed costs of a clinic-based, primary care intervention that integrates a CHW in the role of a PARENT coach into WCVs for children aged 0 to 2 years. Our findings suggest that the intervention was associated with reduced cost of other health care (urgent care, ED visits, hospitalizations, and specialty referrals). We also found that the intervention effect of improved WCV attendance was associated with increased revenue, due to the per-visit payment rate for FQHCs. Depending on a clinic’s visit volume and reimbursement structure, this increased revenue might cover the recurrent intervention costs (CHW salary and benefits).

While the increase in odds of participants using referrals for subspecialty care in the intervention compared with control groups was statistically significant, the decrease in cost of subspecialty referrals among children with visits in the intervention group was statistically significant. It is unclear how the intervention could account for this difference, but 1 post hoc hypothesis is that with a PARENT coach as part of the visit, the primary care clinician can spend less time during the visit focused on providing anticipatory guidance or addressing social needs and more time listening to parents and discussing medical concerns, which may allow them to make more timely and appropriate referrals. This could lead to improved efficiency of referrals.^20^

The decreased odds of participants using other care (urgent care, ED visits, and hospitalizations) were statistically significant for the intervention group, leading to a lower combined total cost of these services among the intervention group participants compared with the control group. Although we did not find a trial effect for ED utilization, our present analysis showed an association with a more comprehensive measure that included urgent care visits and hospitalizations.

Our analysis suggests how FQHCs can leverage the intervention impact on WCV attendance to cover the recurring intervention costs. Clinics and practices that do not receive this PPS rate could not leverage the intervention impact. However, our analysis may have relevance to non-FQHC payment systems, in that the value of increased attendance of WCVs is an important quality metric (eg, Medicaid core set).^21^ For example, payors may consider an enhanced rate per member per month that pays for the PARENT intervention, to help clinics achieve increased WCV attendance.

This study is unique in its methodology; we applied DEX results to analyze non-WCV costs. Estimating the cost per unit of care is challenging, because patients seek care from multiple providers with different billing systems, and costs of care can vary by condition, provider, and health insurer. Previous researchers have estimated the unit cost of outpatient visits and hospital care, but these estimates have not been specific for health condition or patient age.^22,23^ Our study is the first, to our knowledge, to apply DEX estimates in a cost analysis of a health care intervention, using condition and age-specific estimates for costs. DEX provides a rich source of data on unit cost by health condition, age, and year at the national level that will improve cost and cost-effectiveness analyses broadly.

### Limitations

This study has limitations. In the revenue model, we used recurring cost of the intervention (CHW salary and benefits) but excluded the fixed cost of the 6-week CHW training. Our findings are based on DEX data from 2016, but these data can be converted to 2024 US dollars, or any other year of interest, using published conversion factors.

## Conclusions

In this secondary analysis of a cluster RCT, we found that the cost of the PARENT intervention was offset by savings in non-WCV health care use. In addition, FQHCs may be able to recoup some or all of the costs of the PARENT intervention through its impact on WCV adherence.

## Notes

Supplement 1. **eFigure.** Profile of the PARENT Cluster-Randomized Clinical Trial **eTable 1.** Health Care Utilization Questions in the Baseline, 6-Month, and 12-Month Interviews **eTable 2.** Descriptive Statistics on Unit Cost and Frequency of Visits by Age, Service Type, and Condition

Supplement 2.
Trial Protocol

Supplement 3.
Data Sharing Statement
