# Supplementary material for: Cost Analysis of the PARENT Trial of Community Health Workers in Early Childhood Preventive Care: A Secondary Analysis of a Cluster-Randomized Clinical Trial
Source: JAMA Netw Open. 2025 Jul 31;8(7):e2522732. doi: 10.1001/jamanetworkopen.2025.22732 (PMC12314717; doi:10.1001/jamanetworkopen.2025.22732)
Supplement: Supplement 1. — eFigure. Profile of the PARENT Cluster-Randomized Clinical Trial eTable 1. Health Care Utilization Questions in the Baseline, 6-Month, and 12-Month Interviews eTable 2. Descriptive Statistics on Unit Cost and Frequency of Visits by Age, Service Type, and Condition [file jamanetwopen-e2522732-s001.pdf]

Supplementary Online Content

Coker TR, Lowry SJ, Dwibedi E, et al. Cost analysis of the PARENT trial of community health workers in early childhood preventive care: a secondary analysis of a cluster-randomized clinical trial. *JAMA Netw Open.* 2025;8(7):e2522732. doi:10.1001/jamanetworkopen.2025.22732

eFigure. Profile of the PARENT Cluster-Randomized Clinical Trial

eTable 1. Health Care Utilization Questions in the Baseline, 6-Month, and 12-Month Interviews

eTable 2. Descriptive Statistics on Unit Cost and Frequency of Visits by Age, Service Type, and Condition

This supplementary material has been provided by the authors to give readers additional information about their work.

© 2025 Coker TR et al. *JAMA Network Open*.

eFigure. Profile of the PARENT Cluster-Randomized Clinical Trial

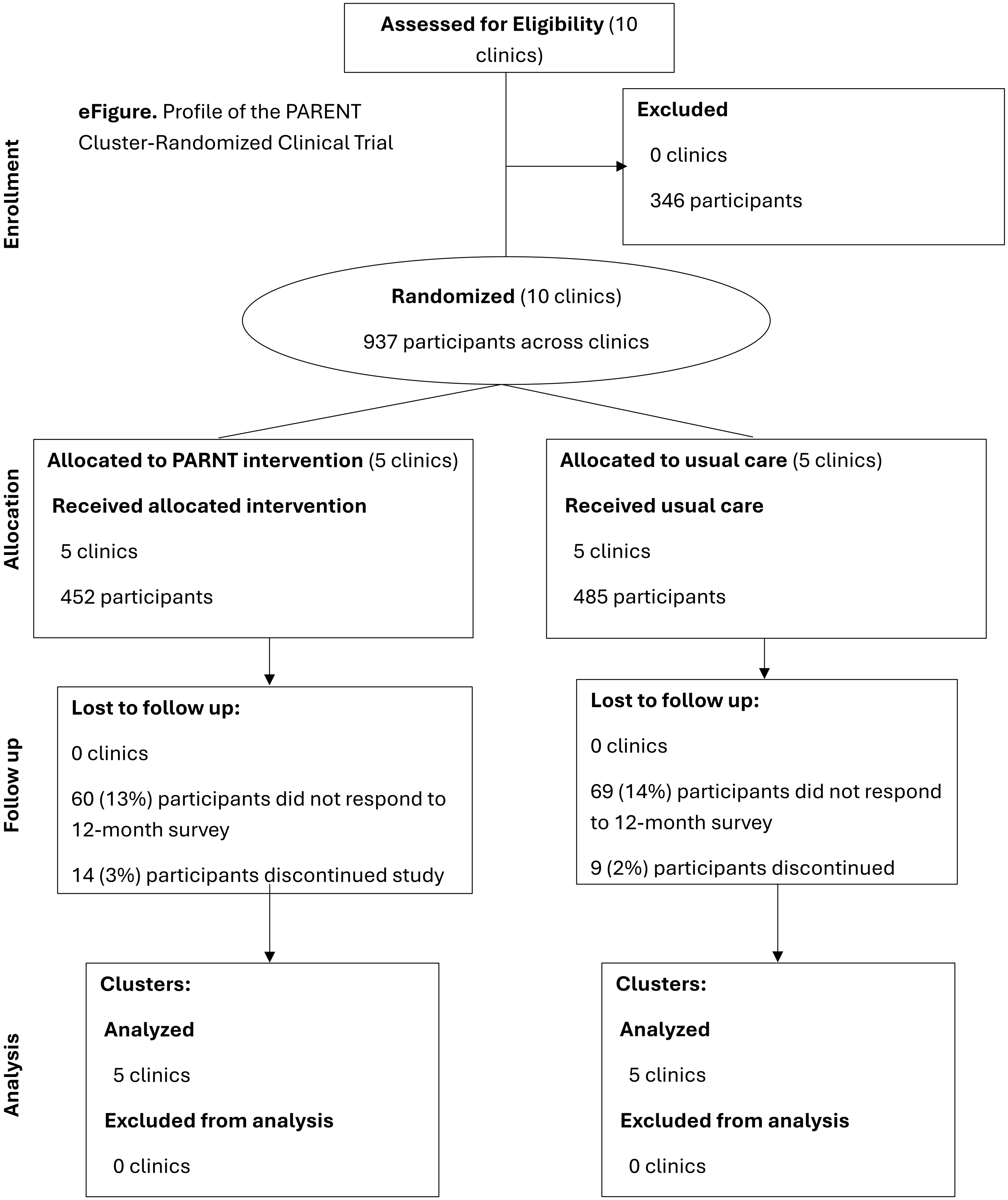

*Reasons include: Parent has a) already seen parent coach; b) not planning to stay at clinic; c) not legal guardian; c) age less than 18 years, d) already enrolled into the study with another child

© 2025 Coker TR et al. *JAMA Network Open*.

eTable 1. Health Care Utilization Questions in the Baseline, 6-Month, and 12-Month Interviews	Response Options
**Emergency Department (ED) Utilization Questions**	
1.1 How many different times did you take your child to a hospital emergency room for emergency care?	Dropdown; numeric

© 2025 Coker TR et al. *JAMA Network Open*.

1.2 Thinking about the emergency room your child visited most recently (if more than one), what is the name of the hospital?	text
1.3 What is the location/address of the hospital?	text
1.4 On what date did this emergency room visit occur?	text (date_mdy)
1.5 What was the main reason your child entered the emergency room? What was the diagnosis?	notes
Probe [as needed]: Ask about symptoms, diagnosis, medications prescribed and procedures completed.	
Questions 1.2 to 1.5 were repeated for each ED visit beginning with most recent.	
**Inpatient Hospitalization Utilization Questions**	
2.1 How many separate overnight hospital stays did your child have?	Dropdown; numeric
2.2 Thinking about the hospital stay your child had most recently (if more than one), what is the name of the hospital?	text
2.3 What is the location/address of the hospital?	text
2.4 On what date did this hospital stay begin?	text (date_mdy)
2.5 How many nights was your child in the hospital for this stay?	Dropdown; numeric
2.6 What was the main reason your child entered the hospital? What was the diagnosis?	notes
Probe [as needed]: Ask about symptoms, diagnosis, medications prescribed and procedures completed.	
2.7 Did the hospital stay include time in the Intensive Care Unit (ICU)?	radio; yes/no)
1.8. If "yes": How many nights was your child in an Intensive Care Unit?	text
Questions 2.2 to 2.8 were repeated for each inpatient hospital stay beginning with the most recent.	
**Urgent Care Utilization Questions**	
3.1 How many times did you take your child to an urgent care center?	Dropdown; numeric
3.2 What is the name of urgent care center you visited most recently (if more than one)?	text
3.3 What is the location/address of the urgent care center?	text
3.4 What was the main reason you took your child to an urgent care center? What was the diagnosis?	notes

© 2025 Coker TR et al. *JAMA Network Open*.

Probe [as needed]: Ask about symptoms, diagnosis, medications prescribed and procedures completed.	
Questions 3.2 to 3.4 were repeated for each urgent care visit beginning with the most recent.	
**Subspecialty Services Utilization Questions**	
4.1 What subspecialty referral services did your child receive? Referral Services List (Check all that apply)	checkbox
4.2 Other (please specify):	text
4.3 How many visits did the child make for the [FIRST] subspecialty referral provider?	text
4.4 How many visits did the child make for the [SECOND] subspecialty referral provider?	text
4.5 How many visits did the child make for the [THIRD] subspecialty referral provider?	text

© 2025 Coker TR et al. *JAMA Network Open*.

eTable 2. Descriptive Statistics on Unit Cost and Frequency of Visits by Age, Service Type, and Condition									
Ages 0 to 11 months					Ages 1 to 4 years				
Service/Condition	Mean unit cost	SD	Coeffi-cient of variation (3)/(2)	Frequen-cy of en-counters	Service/Condition	Mean unit cost	Coeffi-cient of variation (8)/(7)	Frequen-cy of en-counters	Coeffi-cient of variation
(1)	(2)	(3)	(4)	(5)	(6)	(7)	(8)	(9)	(10)
**Subspecialty referrals**					**Subspecialty referrals**				
All condition	$390	69	0.18	78	All condition	$528	46	0.09	116
Congenital anomalies	$529	29	0.05	28	Sense organ diseases	$473	117	0.25	29
Cardiovascular diseases	$11,735	14,131	1.20	22	Endocrine, metabolic, blood, and immune disorders	$245	84	0.34	20
Oral disorders	217	65	0.30	16	Neurological disorders	$2,094	835	0.40	20
Sense organ diseases	$583	159	0.27	13	Urinary diseases and male infertility	$2,063	619	0.30	16
Other non-communicable diseases	$662	100	0.15	8	Digestive diseases	$750	281	0.37	16
Urinary diseases and male infertility	$4,310	2,583	0.60	8	Cardiovascular diseases	$5,424	2,195	0.40	15

© 2025 Coker TR et al. *JAMA Network Open*.

Endocrine, metabolic, blood, and immune disorders	$278	100	0.36	6	Skin and subcutaneous diseases	$648	107	0.17	13
Skin and subcutaneous diseases	$721	129	0.18	4	Congenital anomalies	$529	28	0.05	12
Digestive diseases	$760	144	0.19	4	Oral disorders	$217	65	0.30	2
**Emergency department visits**					**Emergency department visits**				
Upper respiratory tract infections	$672	23	0.03	52	Upper respiratory tract infections	$672	23	0.03	83
All condition	$904	48	0.05	25	Communicable, maternal, neonatal, and nutritional disorders	$760	23	0.03	50
Digestive diseases	$1,536	476	0.31	17	All condition	$905	14	0.02	36
Lower respiratory tract infections	$1,186	42	0.04	16	Otitis media	$537	19	0.03	28
Skin and subcutaneous diseases	$643	35	0.05	11	Falls	$1,129	56	0.05	20
Communicable,	$834	68	0.08	11	Other	$1,05	56	0.05	19

© 2025 Coker TR et al. *JAMA Network Open*.

maternal, neonatal, and nutritional disorders					unintentional injuries	2			
Otitis media	$551	19	0.03	10	Lower respiratory tract infections	$1,165	41	0.03	18
Falls	$1,287	79	0.06	6	Digestive diseases	$996	46	0.05	16
Other unintentional injuries	$1,250	207	0.17	4	Skin and subcutaneous diseases	$628	33	0.05	16
Diarrheal diseases	$1,188	39	0.03	3	Endocrine, metabolic, blood, and immune disorders	$2,135	313	0.15	9
**Ambulatory and urgent care visits**					**Ambulatory and urgent care visits**				
Upper respiratory tract infections	$345	60	0.17	39	Upper respiratory tract infections	$328	56	0.17	98
Skin and subcutaneous diseases	$721	129	0.18	23	Skin and subcutaneous diseases	$648	107	0.17	52
Otitis media	$1,243	327	0.26	13	All condition	$528	46	0.09	42
All condition	$390	69	0.18	12	Communicable, maternal,	$644	97	0.15	35

© 2025 Coker TR et al. *JAMA Network Open*.

					neonatal, and nutritional disorders				
Communicable, maternal, neonatal, and nutritional disorders	$748	236	0.32	9	Otitis media	$1,139	287	0.25	26
Digestive diseases	$760	144	0.19	9	Digestive diseases	$750	281	0.37	20
Lower respiratory tract infections	$347	108	0.31	6	Chronic respiratory diseases	$1,084	169	0.16	12
Sense organ diseases	$583	159	0.27	3	Urinary diseases and male infertility	$2,063	619	0.30	11
Diarrheal diseases	$1,141	76	0.07	1	Lower respiratory tract infections	$301	84	0.28	11
Neonatal disorders	$890	530	0.60	1	Endocrine, metabolic, blood, and immune disorders	$245	84	0.34	4
**Hospital admissions**					**Hospital admissions**				
Congenital anomalies	$133,231	27,295	0.20	3	Lower respiratory tract infections	$12,762	1243	0.10	5
Lower	$12,5	1,191	0.09	2	Communicable,	$13,9	831	0.06	3

© 2025 Coker TR et al. *JAMA Network Open*.

respiratory tract infections	62				maternal, neonatal, and nutritional disorders	55			
All condition	$13,627	956	0.07	1	Other neurological disorders	$19,909	3,236	0.16	3
Urinary diseases and male infertility	$14,851	2,442	0.16	1	Congenital anomalies	$51,818	10,559	0.20	3
Skin and subcutaneous diseases	$22,032	2,390	0.11	1	Asthma	$6,655	717	0.11	1
					Injuries	$23,386	2,253	0.10	1
					Skin and subcutaneous diseases	$11,324	1,219	0.11	1
					Upper respiratory tract infections	$9,283	1,318	0.14	1

© 2025 Coker TR et al. *JAMA Network Open*.
