# Supplementary material for: Cost Analysis of the PARENT Trial of Community Health Workers in Early Childhood Preventive Care: A Secondary Analysis of a Cluster-Randomized Clinical Trial
Source: JAMA Netw Open. 2025 Jul 31;8(7):e2522732. doi: 10.1001/jamanetworkopen.2025.22732 (PMC12314717; doi:10.1001/jamanetworkopen.2025.22732)
Supplement: Supplement 2. — Trial Protocol [file jamanetwopen-e2522732-s002.pdf]

PROJECT TITLE:

Well Child Care Clinical Practice Redesign: A Parent Coach-Led Model of Care for Young Children.
Phase 2: Cluster RCT

PRINCIPAL INVESTIGATOR:

Tumaini R. Coker, MD, MBA
Associate Professor of Pediatrics, Seattle Children's
310-663-6721
Tumaini.coker@seattlechildrens.org

Peter G. Szilagyi, MD, MPH (UCLA PI)
Professor of Pediatrics, UCLA
310-206-6328
Pszilagyi@mednet.ucla.edu

Clinical Trial Registration: clinicaltrials.gov, NCT03797898

Contents

1. Objectives 3
2. Background 3
3. Study Endpoints 4
4. Drugs, Devices and Biologics 5
5. Procedures Involved 5
6. Data and Specimen Banking 9
7. Sharing of Results 10
8. Study Timelines 10
9. Study Population 10
10. Number of Subjects 12
11. Withdrawal of Subjects 12
12. Risks to Subjects 13
13. Potential Benefits to Subjects 13
14. Data Analysis/Management 13
15. Confidentiality 14
16. Provisions to Monitor Data to Ensure the Safety of Subjects 15
17. Use of Social Media 15
18. Research Related Injury 15
19. Recruitment Methods 15
20. Consent/Assent Process 17
21. Process to Document Consent in Writing 20
22. HIPAA Authorization and RCW Criteria 21
23. Payments/Costs to Subjects 23
24. Setting 23
25. Resources Available 23
26. Coordinating Center Procedures 24
27. Good Clinical Practice 24

1. Objectives

1.1. Purpose, specific aims, or objectives:

Purpose:

Conduct a cluster Randomized Controlled Trial (RCT) of a newly designed model of Well-Child Care (WCC) (Phase 2)

Specific aims:

Aim 1: Measure the effect of PARENT on the receipt of nationally-recommended WCC services and parent experiences of care.

Aim 2: Determine the effects of PARENT on WCC, urgent care, and ED utilization, and on net costs.

Aim 3: Examine the effect of PARENT on clinician time allocation for WCC and urgent care visits.

Aim 4: Assess the effect of PARENT on parent-focused outcomes in an exploratory analysis.

1.2. Hypotheses to be tested:

H1: PARENT will improve receipt of WCC services and parent experiences of care

H2a: PARENT will result in improved up-to-date rates for WCC visits and reduced ED utilization

H2b: In a cost/cost-offset analysis, we will demonstrate that direct intervention costs of PARENT are offset by net reductions in ED utilization.

H3: Clinicians will shift time from providing routine WCC services in well-visits to chronic disease management and urgent care.

H4: PARENT will result in improved parental mental health parenting self-efficacy, and positive behaviors.

2. Background

2.1. Relevant prior experience and gaps in current knowledge:

Well-Child Care (WCC) visits for child preventive care during the first three years of life are critical because they may be the only opportunity before a child reaches preschool to identify and address important social, developmental, behavioral, and health issues that could have significant impact and long-lasting effects on children's lives as adults. Despite its potential, multiple studies have demonstrated that pediatric providers fail to provide all recommended preventive and developmental services at these visits and that most parents leave the visit with unaddressed psychosocial, developmental, and behavioral concerns. Further, these missed opportunities are more pronounced for children in low-income families.

A critical problem is that the structure of WCC in the U.S. cannot support the vast array of WCC needs of families. Key structural problems include (a) reliance on clinicians (pediatricians, family physicians, or nurse practitioners) for basic, routine WCC services, (b) limitation to a 15-minute face-to-face clinician-directed WCC visit for the wide array of education and guidance services in WCC, and (c) lack of a systematic, patient-driven method for visit customization to meet families' needs. These structural problems contribute to the wide variations in processes of care and preventive care outcomes, resulting in poorer quality of WCC and perhaps worse health outcomes, particularly for children in low-income communities.

To address the gaps in current WCC this study introduces a new model of care to meet the needs of children in low-income communities: Parent-focused Redesign for Encounters, Newborns to Toddlers (PARENT). PARENT is a team-based approach to care using a health educator ("Parent Coach") to provide the bulk of WCC services, address specific needs faced by families in low-income communities, and decrease reliance on the clinician as the primary provider of WCC services. The Parent Coach provides anticipatory guidance, psychosocial screening/referral, and developmental and behavioral surveillance, screening, and guidance at each WCC visit, and is supported by parent-focused pre-visit screening and visit prioritization, a brief, problem-focused clinician encounter for a physical exam and any concerns that require a clinician's attention, and an automated text message parent reminder and education service for periodic, age-specific messages to reinforce key health-related information recommended by Bright Futures national guidelines.

To assess the efficacy off PARENT we will conduct a cluster randomized controlled trial (RCT). The study will be conducted in partnership with 10 clinics.

There are 2 major phases to this study. In Phase 1 (IRB ID: STUDY00000413), we used a Community Engagement & Intervention Implementation process that has been successful in previous studies to guide the intervention adaptation process, Parent Coach training, practice workflow, and intervention implementation in the practices. In Phase 2 (this protocol), we will conduct a cluster RCT of PARENT to determine its effects on quality, utilization, and clinician efficiency, and its cost/cost-offset. Our community partners include two federally-qualified health centers (FQHC). FQHC #1 has 4 clinics participating in the study and FQHC #2 has 6 clinics participating in the study. The total number of clinics participating in the study is 10 clinics randomized at the clinic level to intervention or control condition. The intervention clinics will implement PARENT for all well-visits through age 2 years at their clinical site, and the control clinics will continue usual care (clinician directed well-visit). 1,000 families will be enrolled at infant age ≤12 months and remain in the study for a period of 12 months. Parents will complete a survey at baseline and at 6 and 12-months post enrollment.

2.2. Relevant preliminary data:[1]

This application is the next phase of a previously approved application (IRB ID: STUDY00000413)

2.3. Scientific or scholarly background:

A pilot randomized controlled trial (RCT) (n=251) of the PARENT intervention in 2 clinics demonstrated robust improvements in parent- reported receipt of WCC services (e.g., psychosocial screening, health education and guidance, and developmental screening, surveillance), experiences of care, and reduced emergency department (ED) visit.

2.4. Prior approvals:

None

3. Study Endpoints[2]

3.1. Primary and secondary endpoints:

Improvement of WCC services (e.g., psychosocial screening, health education and guidance, and developmental screening, surveillance).
Improvement of parent experiences of care

Reduction in emergency department (ED) visits

3.2. Primary or secondary safety endpoints:
This study has minimal risk to participants. There are no safety endpoints.

4. Drugs, Devices and Biologics[3]

4.1. Manufacturer and name of all drugs, devices and biologics:
N/A

4.2. Description and purpose of all drugs, devices and biologics:
N/A

4.3. Regulatory status of all drugs, devices and biologics:[4]
N/A

4.3.1. Drugs or Biologics:
☐ IND Exempt. Explain:[5] ✍Click here to enter text.
☐ IND.

4.3.2. Devices:
☐ IDE Exempt. Explain:[6] ✍Click here to enter text.
☐ Abbreviated IDE / Non-Significant Risk. Explain:[7] ✍Click here to enter text.
☐ IDE / Significant Risk.

4.4. Plans to store, handle, and administer any study drugs, devices and biologics so they will be used only on subjects and be used only by authorized investigators:
N/A

5. Procedures Involved

5.1. Study design:[8]
Our community partners are two federally-qualified health centers (FQHC). FQHC #1 is located in the Tacoma area in Washington and the FQHC #2 is located in the San Fernando/Santa Clarita areas in Los Angeles, CA. FQHC #1 has 4 clinics participating in the study and FQHC #2 has 6 clinics participating in the study. The total number of clinics participating in the study is 10. The cluster randomization was conducted at the end of 2017 by the study statistician. The clinics were placed in blocks by FQHC, location and size and the assignment to control or intervention was done using a computer-generated random allocation. Below are the cluster-randomized assignments, stratified by block and clinic size:

Block	FQHC #1	Assignment
1	Eastside<br>Lakewood	Intervention<br>Control
2	Parkland<br>Spanaway	Control<br>Intervention

Block	FQHC #2	Assignment
1	Canoga Park<br>Van Nuys	Control<br>Intervention
2	Pacoima<br>San Fernando	Intervention<br>Control
3	Santa Clarita<br>Valencia	Intervention<br>Control

The five clinics randomized to intervention will implement the newly adapted model of well child check-up visits for children ages 0-3. The five clinics randomized to control will continue to use the same services that the clinics provide--their usual check-up visit as their typical clinician-led visit (well-visit time slots vary by practice).

Parent/legal guardians will receive the same services offered by these clinics for their child's health care whether they participate in this study or not.

Components of the newly designed model of WCC:

The PARENT intervention was adapted during the Project Working Group meetings in partnership with the two FQHCs (Phase 1: IRB ID: STUDY00000413).

- **Parent Coach at each visit.** The Parent Coach is a Spanish/English-bilingual trained health educator hired by the respective FQHC who received seven weeks of training. The training consisted of self-directed learning based on *Bright Futures Guidelines, Third Edition*, relationship building with community organizations near each clinical site; mock visits; and pediatrician-observed visits with feedback at the practices. The Parent Coach will be available at the intervention clinics to serve as the primary provider of anticipatory guidance, psychosocial screening and referral, and developmental/behavioral guidance and screening at each check-up visit. They will talk to parents about any concerns parents might have about feeding, sleeping, parenting, safety, or other issues. The parent coach will use an adapted version of the *Bright Futures Previsit Questionnaire, Third Edition* for each well-child care visit to guide the visits with the parent. They will document their visit in the child's Electronic Health Record.

- **Well Child Help Line.** The Parent Coach will be available to answer parents' preventive care-related questions, conduct parent follow-up calls and visit reminders during business hours.
- **HealthyText Messages.** Parents will be offered the text messaging service at study enrollment and research staff will process the enrollment by providing the parent's cell phone #, child's DOB and language preference (English/Spanish). The library of text messages (English and Spanish) were adapted from Healthy-TXT, a text messaging service (Healthy-TXT, LLC, Chicago, IL), to the needs of the FQHCs. If parent choose to sign up, they will receive text messages each week focused on age-appropriate anticipatory guidance, health education, and reminders for WCC visits. Most messages include a link to an educational website (e.g., healthychildren.org) with a video or written information on that specific topic, or the message may include the clinic's telephone number for visit scheduling, the parent coach number or other information (e.g., poison control hotline). At any time, parents will be able to text STOP to end the service.

5.2. Research procedures:[9]

Intervention and Control clinics: Parents/legal guardians checking in for a WCC visit or a visit in between for an infant ≤12 months will be approached by research staff. Research staff will obtain a schedule of WCC visit prior to visiting the clinic. The schedule will include date, type of WCC visit, language, MRN, and child's age in weeks or months, parent name, parent phone number, and home address.

Parent/ legal guardians will be approached while waiting for their WCC visit or by phone if research staff are unable to approach in-person.

Clinic flyer procedure:
Clinics will have flyers on display about the study as a method for alerting parents to the possibility of being approached by research staff. Parents may take a flyer from the clinic to initiate communication with study staff to determine eligibility, learn more about the study, review the information sheet, and give verbal consent.

Clinic recruitment procedure:
Research staff will let the clinic staff and front desk staff know when arriving and verify the schedule. The front desk staff will let parents/legal guardians know that someone is at the clinic to talk to them about the research study. If parent/legal guardian agrees to be approached, the front desk staff will let the research staff know.

Research staff will explain the study, screen for eligibility (they will be asked some questions), provide the parental permission and consent form, and obtain consent. When possible, parents/legal guardians will be given the opportunity to discuss the study in a private room.

Research staff will conduct the baseline survey after screening and obtaining consent while parent/legal guardian is waiting for their WCC visit in the waiting area. We will assign IDs to all screened parents/legal guardians but will conduct the survey only with those wanting to participate and if they are eligible. If the survey is not completed during the waiting room time period, we will ask parent/legal guardian if it is possible to complete the survey at the end of their WCC visit or by phone the same day or any other day but not later than 14 days from date of enrollment.

Phone call recruitment procedure:
When research staff are limited from recruitment on-site, clinic sites will be informed of pre-screened visits that are anticipated to be eligible for enrollment.

CHC and NEVHC sites:
1. Clinic Staff approach in-person: When clinic staff are available in-person to communicate with the parent/legal guardian of a pre-screened patient (pre-screened by the research team), the clinic staff will give a physical copy of the information sheet, and will ask if the research team can call the parent/legal guardian to explain the study, screen for eligibility, review the information sheet, and obtain verbal consent. Parents/legal guardians will have the option to decline a call from the research team by letting clinic staff know they do not want to be

contacted. Clinic staff will communicate with the research team to let them know which families were approached by clinic staff and agreed or declined to receive a call from the research team.

2. Clinic staff approach over the phone: When clinic staff conduct pre-visit calls by phone to review appointment procedures with a parent/legal guardian, the clinic staff will communicate with the parent/legal guardian of a pre-screened patient (pre-screened by the research team) to inform them of the study. The clinic staff will ask if the research team can call the parent/legal guardian to explain the study, send the parent an information sheet, screen for eligibility, review the information sheet, and obtain verbal consent. Parent/legal guardians will have the option to decline a call from the research team by letting clinic staff know they do not want to be contacted. Clinic staff will communicate with the research team to let them know which families were approached by clinic staff and agreed or declined to receive a call from the research team.

Additional recruitment method for CHC patients:
1. Parent/legal guardians of a pre-screened patient (pre-screened by the research team) will be mailed an (i) opt-out letter co-signed by CHC's medical director and Study PI and an (ii) information sheet. Parent/legal guardians will have the option to decline a call from research staff by responding to the letter by email, call, or text. Research staff will call to explain the study, screen for eligibility, review the information sheet, and obtain verbal consent.

Additional recruitment procedure details:
For those approached by phone, study staff who have access to the clinic WCC visit schedule and that have received training to use the clinic's EHR will document the consent process by entering the date and time in RedCap.

We will collect information on the number of people approached, the number of people who agreed to participate and the number of people who refused to participate and the reasons for their refusal.

We will collect contact information from the enrolled parent/legal guardian and spouse/partner (home and cell phone, email) to telephone them to complete the baseline survey, if needed. The information will be verified at each subsequent contact for the follow up surveys at 6-month and 12-months from date of enrollment. We will also ask parents to provide contact information for three relatives/friends who will always know their whereabouts. Because this study population frequently changes phone numbers, we will also ask participants to provide any social media handles, if they are willing, so that the study team can direct message them. Direct messaging will be utilized to get updated phone numbers or schedule a phone conversation and no other purposes. Additionally, to support retention we will contact participants by cell phone and email at 3- and 9-month time points to remind them of their study participation as well as confirm their contact information has not changed. Furthermore, we will send participating families a card for their baby's first birthday and for New Years from the research team to encourage engagement, remind them of their participation, and support follow-up survey retention.

The research staff will ask parents/legal guardians questions about their experiences in getting care so that we can compare the two types of well-child care.
We will conduct three waves of survey data (baseline, 6-month and 12-month) collection and chart review at the end:
- Baseline Survey (at enrollment): Experienced research staff will be responsible for data collection. Upon enrollment, parents will participate in a 35-minute survey to collect baseline demographic data on the infant, parent, and household and child's medical history.

• 6-Month Survey (participant retention survey by phone): Research staff will conduct a 15-minute phone survey at 6-months post-enrollment to aid in participant retention. The 6-month survey will update the child's medical history, household changes, and healthcare utilization (e.g., ED utilization).
• 12-month Survey: At 12-months post-enrollment, all participants will be asked to complete a 40-minute survey by phone. This survey administration will include the Promoting Healthy Development Survey (PHDS-PLUS), a parent survey that assesses the provision of nationally recommended WCC services. We will use the PHDS-PLUS to determine parent perceived receipt of recommended WCC services, appropriate follow-up, and health care utilization. Additional questions on social determinants screening and referral are included and drawn from the PHDS-PLUS as well as the National Survey of Early Childhood Health. We will also use items on overall satisfaction of care from the Consumer Assessment of Healthcare Providers and Systems (CAHPS) Health Plan Survey and family-centeredness of care from the National Survey of Children's Health. Using previously validated parent survey tools, we will collect data on our exploratory measures of parental mental health, perceived parenting self-efficacy and parenting behaviors.
For the 6-month and 12 month follow up surveys, we will contact study participants two weeks before the due date. If we are unable to reach them by the due date, we will continue calling them up to three months after the due date to complete the survey.
• Chart review: After completing the three waves of survey data collection, we will conduct chart review. The chart review includes clinic visits, ED visits, services received at those visits, immunizations, and status of referrals.

5.3. Data sources that will be used to collect data about subjects:[10]

Parent-report surveys and chart review from electronic medical records. All surveys, scripts, and data collection forms are attached in the Supporting Documents page

5.4. Data to be collected, including long–term follow–up data:[11]

Baseline Survey (at enrollment), 6-Month Survey (participant retention survey) and 12-month Survey and Chart review at 12-month post enrollment.

6. Data and Specimen Banking[12]

6.1. Complete list of the data and/or specimens to be included in the bank:[13]

Names and contact information for participants enrolled prior to March 2020. Participant baseline data prior to March 2020.

6.2. Location of data and/or specimen storage:[14]

RedCap and WCC Redesign study shared drive

6.3. List of those with direct access to data and/or specimens in the bank:

Study staff who are both on the WCC Redesign project and the COVID Survey Study (STUDY00002666, approved by the SCH IRB on 07/01/20).

6.4. Length of time data and/or specimens will be stored in the bank:

Data will not be stored in a separate bank but will be accessed only by the COVID Survey Study for the length of that study.

6.5. Procedures for protecting the confidentiality and privacy of the subjects from whom the data and/or specimens were collected:[15]

Only study team members who currently have access to the information as part of the WCC Redesign study will access information for the purposes of COVID Survey Study recruitment. Contact information will be accessed, per the appropriate waivers (approved for the COVID Survey Study). Only WCC Redesign data from those who consent for enrollment in STUDY00002666 will be accessed.

6.6. How the data and/or specimens will be made available for future use:
Only relevant research staff will have access to the data.

6.6.1. Who can request data and/or specimens from the bank:
Only the COVID Survey study will be accessing this data.

6.6.2. Format in which data and/or specimens will be provided:
The COVID Survey Study staff will have full access to the RedCap data and participant contact information as it is stored in the study shared drive. The same staff works on both projects.

6.6.3. Process for investigators to request data and/or specimens:[16]
Use of the data from the WCC Redesign Study is limited to the COVID Survey Study and the same study staff are on the two studies, so no process to request data has been established.

6.6.4. Restrictions on future use:[17]
No other studies are being given access to this study data at this time.

6.6.5. Plan for providing data results from banked data/specimens:
Any plan to share results would be covered within the COVID Survey Study protocol.

7. Sharing of Results

7.1. Plan to share results with subjects/others:[18]
The results of the evaluation will be shared with participants using a flyer. The results will be presented only in aggregate form and with data that is de-identified. We will not put any data in the child's medical record

8. Study Timelines

8.1. Duration of an individual subject's participation in the study:
12 months

8.2. Duration anticipated to enroll all study subjects:
12 months

8.3. Estimated date for the investigators to complete this study:
June 2021

9. Study Population[19]

9.1. Inclusion criteria for each subject population (e.g., patients, parents, providers):
Parent/legal guardian-Child dyad attending well child check-up visits or visits in between for a newborn to 12-month.

Parent is English or Spanish proficient
For multiple gestations, one infant will be randomly selected as the index child. Infants with special health care needs will not be excluded from the study, since these children generally need the same recommended preventive care services.

9.2. Exclusion criteria for each subject population:

More than one child attending WCC
Legal guardian of child is under 18 years of age
Parents who are employed by the CHC/NEVHC
Either member of the dyad previously enrolled
Sick-visit
Parents/legal guardian planning not to continue receiving well child care services at this clinic for their child in the next 12 months

9.3. Vulnerable populations involved in the study:[20]

☒ Children/Teenagers[21]

Risk assessment specific to this vulnerable population and additional safeguards:[22]

This study does not involve direct interaction with children but involve minimal interactions with the parents/legal guardians of the children attending well-child care visits to complete the study surveys. The minimal risk status of this study does not indicate the need for additional safeguards for these populations.

☐ Children who are Wards of the State[23]

Risk assessment specific to this vulnerable population and additional safeguards:

✍Click here to enter text.

☐ Adults Unable to Consent[24]

Risk assessment specific to this vulnerable population and additional safeguards:

✍Click here to enter text.

☐ Neonates of Uncertain Viability or Non–Viable Neonates[25]

Risk assessment specific to this vulnerable population and additional safeguards:

✍Click here to enter text.

☒ Pregnant Women[26]

Additional safeguards:

This study involves minimal interactions with pregnant women who might be the parents/legal guardians of the children attending well-child care visits to complete the study surveys. The minimal risk status of this study does not indicate the need for additional safeguards for these populations.

☐ Prisoners[27]

Additional safeguards:

✍Click here to enter text.

☒ Economically or educationally disadvantaged persons[28]

Additional safeguards:

This study involves direct interaction with economically or educationally disadvantaged persons and the following safeguards aid to minimize coercion or undue influence. (1) The recruitment process at the control and intervention sites have been designed to ensure participation is voluntary. (2) Recruitment materials do not promise “free” services or treatment, nor do they emphasize direct benefit to the participating subject during the research. (3) Incentive for research participation are commensurate with the risk, discomfort, and inconvenience involved in research, and financial or gains are not overly compelling. (4) Consent documents are written in language that is understandable to participants. (5) Study materials have been designed for the possibility of limited reading skills and the need to communicate in a foreign language (e.g., Spanish). (6) Participant will be reminded of their rights as a participant to ensure open and free communication between researcher and the prospective subject. The minimal risk status of this study does not indicate the need for additional safeguards for these populations.

10. Number of Subjects

10.1. Total number of subjects to be enrolled locally:[29]

100 parent/legal guardian-child dyads per each of the four clinics in Tacoma, WA. For a total of 400.

10.2. Total number of subjects to be enrolled across all participating sites:[30]

100 parent/legal guardian-child dyads per each of the 10 clinics. For a total of 1,000.

10.3. Number of screened subjects versus the actual number enrolled in the research:[31]

2 to 1. In other words, we will expect to screen doubled the number of actual people enrolled in the study

10.4. Power analysis:

The power calculation is based on WCC quality (anticipatory guidance) and ED visits (2 or more), with a 1:1 randomization of 10 sites into the intervention and control groups. We use the mean and standard deviation (SD) of anticipatory guidance and the rates of 2 or more ED visits as observed in our pilot RCT. For anticipatory guidance, we use the larger SD from the two groups to have a conservative power estimation for this variable. The calculation assumes an intra-class correlation of 0.01 based on previous delivery systems design cluster RCTs among similar populations[81, 82] and the fact that the data analysis will be adjusted for important baseline covariates when comparing the intervention versus control groups[83]. A 20% dropout rate is assumed at the patient level. Two-sided tests are used with a type I error rate of 0.05. With an N of 75 per site, we will have at least 80% power to detect the intervention effect for both anticipatory guidance and ED visits. After accommodating a 20% dropout rate at the patient level, the N per site is 94, or a total sample of 940 participants.

11. Withdrawal of Subjects

11.1. Anticipated circumstances under which subjects will be withdrawn from the research without their consent:

Subjects can choose whether to be in this study or not. If they agree to participate, they may withdraw their consent at any time without impacting their care. For this study, we do not anticipate the need to withdraw subject due to investigator judgement.

11.2. Procedures for orderly termination:
N/A

11.3. Procedures that will be followed when subjects withdraw from the research, including partial withdrawal from procedures with continued data collection and withdrawal from data/specimen banking:
Data collected on any withdrawn subjects will be removed from the analysis

12. Risks to Subjects

12.1. Reasonably foreseeable risks to subjects (include each study population, each arm, and optional procedures):
There is a risk that confidentiality or privacy could be breached and people could find out if someone was in the study.

12.2. Procedures with unforeseeable risks:
N/A

12.3. Procedures with risks to an embryo or fetus should the subject be or become pregnant:
N/A

12.4. Risks to others who are not subjects:
N/A

12.5. Procedures performed to lessen the probability or magnitude of risks:
N/A

13. Potential Benefits to Subjects

13.1. Potential benefits that individual subjects may experience from taking part in the research:[32]
We do not anticipate any direct benefit to participating subjects.

14. Data Analysis/Management

14.1. Data analysis plan, including statistical procedures:
We will compile descriptive statistics on all outcome variables, composite scores, and covariates. We will report means, medians, and standard deviations for continuous variables, create graphical displays to visualize distributions, and transform variables with non-normal distributions. Next, we will use multivariable analyses to examine differences between the control and intervention groups on the measures described above. From these analyses, we will estimate intervention effects. An intent-to-treat approach will be used, that is, parents will be analyzed according to the assigned group regardless of deviations from the study protocol. Sensitivity analysis will be conducted by performing the analyses with and without outliers and influential data points. Missing values will be excluded from the analysis if the missing rate is low (< 10%). If the missing rate is more than 10%, data may also be imputed using multiple imputation techniques and the analysis can then be done based on the complete, imputed datasets. All tests are two-sided, and p values < 0.05 are considered statistically significant.

Specific Aim 1: We will analyze anticipatory guidance based on data collected at 12-months post enrollment. We will assess intervention effect on anticipatory guidance via linear mixed effects models that involve random effects to capture data clustering within clinic, given

the fact that randomization is done at the clinic level. The outcome may be subject to transformation prior to fitting the model if non-normality is observed. The analysis will include intervention status as the main independent variable, adjusted for baseline covariates and other confounding factors. We will also test possible interaction effects between intervention and these covariates. All other outcome variables to measure experiences of care and receipt of other nationally-recommended WCC services are regarded as secondary. Each outcome will be analyzed using linear or generalized linear mixed effects models depending upon data distributions, with a similar setting as described above for anticipatory guidance.

Specific Aim 2a: Intervention effects on healthcare utilization (ER visits, hospitalizations, urgent care visits, regional center/early intervention, maternal mental health, support groups (e.g., breastfeeding, parenting classes), dentist, case management/social services) will be assessed using generalized linear mixed effects models for binary or count data with a similar setup as outlined for Aim 1. The model will include random effects to capture data clustering within clinic and the primary independent variable is intervention status. The analysis will be adjusted for any baseline child, parent, or household characteristics that are significantly different between control and intervention groups. We will also test interaction effects between intervention and the covariates.

Specific Aim 2b: Cost/cost-offset Analysis. We will conduct our analysis from the perspectives of the "social planner"80 and of the payer (e.g., health plan or medical group), and consider intervention costs (direct and indirect) and cost offsets.

Specific Aim 4: We will utilize the same approach as described for Specific Aims 1 and 2a; i.e., linear or generalized linear mixed effects models, depending on the distribution of the outcome variables.

14.2. Quality control procedures for collected data:[33]

The study team member who collected the data will enter the data into the study database and another team member will be validating the data entered.

15. Confidentiality[34]

15.1. Procedures to secure the data and/or specimens during storage, use, and transmission:

All electronically maintained data will exist on encrypted devices and secure databases.

15.2. Location where the data and/or specimens will be stored:

Parent/Child participant data will be entered directly into a redcap database on password-protected laptops and smart tablets. Data initially collected on hard copy will be transferred to the secure, database. Hard copy documents will be kept in locked cabinets in a locked office. Identifiable information, such as consent forms, will be kept separately from data collection forms to ensure the de-identification of the data. Hard copy documents that are collected from clinics and transported to study offices will be kept in secure document holders to protect against breeches of confidentiality.

15.3. Length of time data and/or specimens will be stored:

5 years

15.4. Individuals with access to data and/or specimens:

Only relevant research staff will have access to the data.

15.5. Process for the transmission of data and/or specimens outside Seattle Children's:

15.5.1. List of data and/or specimens that will be transmitted:

Only de-identified data for purposes of data analysis

15.5.2. Individual(s) who will transmit data:
Only the PI, project manager, or project CRA

16. Provisions to Monitor Data to Ensure the Safety of Subjects[35]

16.1. Plan to periodically evaluate the data collected regarding both harms and benefits to determine whether subjects remain safe:[36]

Research does not involve greater than minimal risk

16.2. Data reviewed to ensure safety of subjects:

N/A

16.3. Safety information collection procedures:

N/A

16.4. Frequency of cumulative data review:

N/A

16.5. Conditions that trigger an immediate suspension of the research:

N/A

17. Use of Social Media

17.1. Types of social media to be used and how:

Twitter, Instagram and Facebook will be used. The only features of these social media platforms that will be used will direct messaging.

17.2. Measures in place to protect the privacy or confidentiality of subjects:[37]

The study social media accounts will never “friend”, follow or engage in any way with participants’ social media pages. The only interaction or engagement will be via direct message. We will not search for individuals on social media and will only direct message those who provide us with permission to do so as well as their specific profile name. Additionally, the study social media accounts will not be public to avoid any possibility of public interactions that could reveal identities of potential participants to each other.

17.3. Types of communications that will be submitted to the IRB for review:[38]

A direct message script will be submitted for review. This script shows the communication will be limited to scheduling and that participants will be encouraged to call the study team to discuss details and/or ask questions.

17.4. If user-generated content will be active, how it will be monitored and what actions will be taken to ensure subject safety and study integrity:

N/A

18. Research Related Injury[39]

18.1. Available compensation in the event of research related injury:

The study does not involve more than minimal risk to subjects

19. Recruitment Methods[40]

19.1. When, where, and how potential subjects will be recruited:

On-site recruitment:
Parents/legal guardians checking in for a newborn through 12 month visit will be approached by front desk staff. Those who agree will be approached while waiting for their WCC visit. Clinics will have flyers on display about the study as an additional method for alerting parents to the possibility of being approach by research staff. For those approached in clinic, study staff will explain the study, screen for eligibility, provide the consent form, and obtain consent. When possible, parents/legal guardians will be given the opportunity to discuss the study in a private room.

Off-site recruitment:

- **Method 1: When research staff are limited from recruitment on-site**: clinic sites will be informed of pre-screened visits that are anticipated to be eligible for enrollment. Clinic staff will be able to give parent/legal guardians a copy of the consent form/information sheet prior to research staff contacting them. Parent/legal guardians will have the option to decline a call from research staff. Study staff (who have access to the clinic WCC visit schedule and that have received training to use the clinic's EHR) will call families to explain the study, screen for eligibility, and obtain verbal consent over the phone and document the consent process by entering the date and time in RedCap.

- **Method 2: Under extenuating circumstances, such as the 2019- 2020 SARS-CoV-2 (COVID-19) for the Tacoma, Washington and Los Angeles, California sites**: Clinic staff will ask parent/legal guardians, over the phone during appointment reminder calls, if the research team can contact them to explain the study. Due to the risks associated with COVID-19 transmission, clinic staff are limited in their ability to provide the study information sheet in-person. Parent/legal guardians will have the option to decline a call from research staff. Parent/legal guardians will only be contacted by research staff after clinic staff inform the parent of the study. Under these specific circumstances, the research staff cannot rely on mailing an opt-out letter and information sheets prior to the patients scheduled visit. The research and clinic staff have no other possible alternative to communicating with participants without increasing the risk to parent/legal guardians and their infant children during this fast-evolving public health crisis.

- **Method 3: To increase accrual rates at Community Health Care (Tacoma, WA) study sites**: Study staff will mail an (i) opt-out letter and the study (ii) information sheet to families that have a clinic visit scheduled or due within a 1-month period for routine care. Parents who receive mail communication from the research team will not be added the pre-screened list that clinic staff inform of the study during their appointment reminder calls. Parent/legal guardians will have the option to decline a call from research staff by replying by call, text, or email. Study staff will call participants about one week after mailings are sent and before the scheduled well-child care visit. The opt-out letter is co-signed by CHC's medical director and the study PI.

19.2. Steps that will be taken to protect potential subjects' privacy interests:[41]

Any information that is obtained in connection with this study and that can identify potential subjects will remain confidential and will be disclosed only with permission of the subject or as required by law. Only the study staff will see the data. This information will not be linked to any names. For participants who choose to provide contact information to complete their survey over the phone their contact information will be temporarily linked to their study ID. A master

log with study ID, name and contact information will be maintained on a secure computer and accessible only by study staff. Once the study has been completed, this link will be destroyed.

19.3. Sources of subjects:[42]
Clinic schedule of visits.

19.4. Methods that will be used to identify potential subjects:
The FQHCs will share a weekly and monthly schedule of their well-child care (WCC) visits per clinic with the date, time, and type of WCC visits, MRN, primary language, and age in weeks or months.

19.5. Materials that will be used to recruit subjects:[43]
Clinics will have flyers on display

19.6. Recruitment methods not controlled by Seattle Children's:
N/A

20. Consent/Assent Process

20.1. Where the consent process will take place:

For those approached for the first time in clinic: study staff will provide the consent form, explain the study, screen for eligibility, and obtain consent. When possible, parents/legal guardians will be given the opportunity to discuss the study in a private room.

Families will be approached by phone when research staff are unable to attend clinic visits due to extenuating circumstances.

CHC and NEVHC sites phone process:
Clinic staff will ask parent either in-person or by phone if a research staff member can call them to explain the study. For those asked in-person the clinic staff will give the parent an information sheet prior to the research staff call (recruitment method 1, listed in section 19.1). During extenuating circumstances, such as the risk for COVID-19 transmission, study staff will not give participants an information sheet. Parents who are approached by clinic staff and agree to be called by the research team will receive an information sheet at the time of the call with the research staff member. The research staff member will send the information sheet to the parents through their preferred method (email or text) to review in real-time with the research staff (recruitment method 2, listed in section 19.1). Research staff will explain the study, screen for eligibility, review the information sheet, and obtain verbal consent. Date and time for parent/legal guardian verbal consent will be documented in RedCap.

CHC site mail and phone process: Pre-screened families will be mailed an opt-out letter and an information sheet (recruitment method 3, listed in section 19.1). Research staff will call and explain the reason for calling in further detail, provide families with study information over the phone (including text or email if requested), screen for eligibility, and obtain verbal consent. Date and time for parent/legal guardian verbal consent will be documented in RedCap.

20.2. Steps that will be taken to protect prospective subjects' privacy interests:[44]
Any information that is obtained in connection with this study and that can identify prospective subjects will remain confidential and will be disclosed only with permission of the subject or as required by law. Only the study staff will see the data. This information will not be linked to any names. For participants who choose to provide contact information to complete their survey

over the phone their contact information will be temporarily linked to their study ID. A master log with study ID, name and contact information will be maintained on a secure computer and accessible only by study staff. Once the study has been completed by phone, this link will be destroyed.

20.3. Waiting period available between approaching a prospective subject and obtaining consent:

Time required to explain the study, screen for eligibility and answer any questions the parent/legal guardian might have.

20.4. Process to ensure ongoing consent:

At each study contact (6 and 12 month survey) we will ask the participant if they would like to continue with the study, reminding them that they may stop at any time.

20.5. If this box is checked, "SOP: Informed Consent Process for Research (HRP-090)" will be followed: ☒

20.6. If "SOP: Informed Consent Process for Research (HRP-090)" will <u>not</u> be followed, address the following:[45]

20.6.1. Role of the individuals listed in the application as being involved in the consent process:

N/A

20.6.2. Time that will be devoted to the consent discussion:

N/A

20.6.3. Steps that will be taken to minimize the possibility of coercion or undue influence:

N/A

20.6.4. Steps that will be taken to ensure the subject's understanding:

N/A

20.7. Non-English Speaking Subjects[46]

20.7.1. Anticipated preferred language(s) for subjects or their representatives:

Spanish

20.7.2. Presentation of Research Information and Documentation:

☒ Appendix A-10 of the Investigator Manual will be followed[47]

☐ Short form procedures may be used per HRP-091. If so, choose applicable box(es):

☐ Per section 5.5.1

☐ Per section 5.5.2

☐ Appendix A-10 of the Investigator Manual will <u>not</u> be followed. Explanation of procedures not following Appendix A-10:

20.7.3. Justification if non-English speaking subjects will <u>be excluded</u> from the research:[48]

N/A

20.8. Subjects Who Are Not Yet Adults (Infants, Children, Teenagers)

20.8.1. Process used to determine whether an individual has not attained the legal age of consent under the applicable law of the jurisdiction in which the research will be conducted (e.g., individuals under the age of 18 years):[49]

All of the children in this study will be infants and will be unable to provide consent

20.8.2. Parental permission will be obtained from:[50]

☐ Both parents unless one parent is deceased, unknown, incompetent, or not reasonably available, or when only one parent has legal responsibility for the care and custody of the child.

☒ One parent even if the other parent is alive, known, competent, reasonably available, and shares legal responsibility for the care and custody of the child.

☐ Neither parent.[51]

20.8.3. Process used to determine an individual's authority to consent to each child's general medical care if permission will be obtained from someone other than parents:[52]

We will ask during the eligibility screening whether or not they are the parent of legal guardian of the child

20.8.4. Assent will be obtained from:[53]

☐ All children.

☐ Some children. Specify:

☒ None of the children. Explain: Assent will not be obtained from children because they are too young to provide it

20.8.5. Procedures for obtaining and documenting assent:

N/A

20.8.6. Plan for re-approaching children who have reached the age of majority to obtain consent:[54]

N/A

20.9. Cognitively Impaired Adults/Adults Unable to Consent[55]

20.9.1. Process used to determine whether an individual is capable of consent:

N/A

20.9.2. Individuals from whom permission will be obtained in order of priority:[56]

N/A

20.9.3. Assent will be obtained from:

☐ All of these subjects.

☐ Some of these subjects. Specify: ✍Click here to enter text.

☒ None of these subjects. Explain: N/A

20.9.4. Process for obtaining and documenting assent:[57]

N/A

20.10. Waiver or Alteration of Consent Process

20.10.1. Reasons for requesting a waiver or alteration of informed consent:[58]

We are requesting a waiver of consent to release information for purposes of recruitment for the COVID Survey Study (STUDY00002666).

20.10.2. Consent Waiver/Alteration Criteria justifications:[59]

20.10.2.1. The research involves no more than minimal risk to the subjects because:

This study involves minimal interactions with the parents/legal guardians of the children attending well-child care visits to complete the study surveys. Chart review will also be conducted. Appropriate confidentiality measures are in place.

20.10.2.2. The waiver or alteration will not adversely affect the rights or welfare of the subjects because:[60]

While participants did not provide consent for their information to be used for another study, it is not precluded by the consent form. The study team conducting the COVID Survey Study are also on the current study, so information will not be shared with new individuals. Participants will be consented before their data (beyond recruitment information) is used for the COVID Survey Study.

20.10.2.3. The research could not practicably be carried out without the waiver or alteration because:[61]

Only information necessary for recruitment is being released prior to consent. Without the information, the study team would not be able recruitment for the COVID Survey Study.

20.10.2.4. Whenever appropriate, the subjects will be provided with additional pertinent information after participation:

Participants will provide consent for the COVID Survey Study prior to data beyond recruitment information being released.

20.10.3. If the research involves a waiver of the consent process for emergency research, provide sufficient information for the IRB to make it determinations:[62]

N/A

21. Process to Document Consent in Writing

21.1. If consent will be documented in writing (check one):

☒ "SOP: Written Documentation of Consent (HRP-091)" will be followed.

☒ "SOP: Written Documentation of Consent (HRP-091)" will not be followed.
Process of documenting consent:[63]

During periods when we are unable to have in person contact with potential participants due to the health risk (COVID-19) SOP: written documentation of consent HRP-091 will not be followed. During these periods consent will be conducted and obtained over the phone and documented by the consenting research staff via RedCap.

During periods when we are able to have in person contact with potential participants SOP: Written Documentation of Consent HRP-091 will be followed.

21.2. If consent will not be documented in writing (check all boxes that apply):[64]

- ☒ A written statement/information sheet describing the research will be provided to subjects.[65]
- ☐ A written statement/information sheet describing the research will not be provided to subjects. Explain: ✍Click here to enter text.
- ☐ A consent script will be used.[66]

22. HIPAA Authorization and RCW Criteria

22.1. HIPAA Authorization (check all boxes that apply):

- ☐ The study does not involve the receipt, creation, use and/or disclosure of protected health information (PHI).[67]
- ☒ HIPAA authorization will be obtained as part of a signed consent form.
- ☒ The study will access PHI without prior authorization from subjects (including for recruitment purposes – e.g., reviewing the medical record to determine eligibility). *See 21.2 below for required HIPAA waiver/alteration criteria*.
- ☒ Subjects will review a written statement/information sheet with the appropriate HIPAA language but will not provide a written signature. See 21.2 below for required *HIPAA alteration criteria*.[68]
- ☒ Other. Explain:[69]
  Study staff will access EHR contact information (including child's MRN, primary language, and visit type, parent name, parent phone number, and home address) prior to consent, for recruitment purposes only, to contact parent/legal guardians that meet preliminary screening (their child is schedule for a clinic visit between the ages of infant ≤12 months, schedule at a participating clinic, and their primary language is English or Spanish).

  We are also requesting a Waiver of HIPAA Authorization to use PHI for the purposes of recruitment for the COVID Survey Study.

22.2. HIPAA Waiver/Alteration Criteria: Explain why:

22.2.1. The use or disclosure of PHI involves no more than a minimal risk to privacy of individuals, based on, at least the presence of the following elements:

22.2.1.1. An adequate plan to protect the identifiers from improper use and disclosure:

-For periods when in person recruitment is prohibited:
Per section 15 all PHI will be collected, stored and disclosed securely utilizing redcap and secure shared folders only accessible to study staff.

For recruitment purposes:
A representative from the FQHC will share the upcoming week and month schedule of WCC visits from all the participating clinics with a research staff member through an encrypted email.

The schedules will be stored electronically in secured drive (e.g., SCH network)
Only trained research staff member will have access to medical records of enrolled participants.

22.2.1.2. An adequate plan to destroy identifiers at earliest opportunity consistent with conduct of research:

Identifiers will be destroyed at the conclusion of all study procedures and data analysis.

22.2.1.3. Assurances that PHI will not be reused or disclosed to any other party or entity, except as required by law or for authorized oversight of the research:

We provide assurance that PHI will not be reused or disclosed to any other party or entity, except as required by law or for authorized oversight of the research.

22.2.2. The research could not practicably be conducted without the waiver or alteration of authorization:

Due to COVID-19 restrictions of in-person contact and on site work for research staff obtaining signed HIPAA authorization is often not possible or highly burdensome to potential participants. In order to continue enrolling participants in this project during periods of required social distancing an alteration of HIPAA authorization is necessary.

For recruitment purposes:
Parents bring their kids to the clinics for all types of reasons and since we are particularly interested in WCC visits and visits in between. Without the schedule of WCC visits, we would not be efficient with our time and resources trying figuring out who to talk to at the clinics.

COVID Survey Study:
While participants did not provide authorization for their information to be used for another study, it is not precluded by the consent form. The study team conducting the COVID Survey Study are also on the current study, so information will not be shared with new individuals. Participants will provide authorization before their data (beyond recruitment information) is used for the COVID Survey Study.

22.2.3. The research could not practicably be conducted without access to and use of the PHI:[70]

For periods when in person recruitment is prohibited:
In order to communicate, track and collect data on study participants PHI must be accessed and used.

For recruitment purposes because:
WCC visits are the focus of the study

WCC visits are age-specific and we are recruiting parent/legal guardian-Child dyad attending WCC visits for a newborn to 12-month.

COVID Survey Study:
In order to identify eligible participants, the study team will need to access data to determine which participants have baseline data.

23. Payments/Costs to Subjects[71]

23.1. Amount, method, and timing of payments to subjects:[72]

Parent/legal guardian will receive a gift card after completing the baseline survey ($30) and the follow-up surveys at 6 months ($20) and 12 months ($40) post enrollment.

Parents/legal guardians will receive an e-gift card, during extenuating circumstances (i.e. COVID-19 pandemic), where business-mailing capabilities are reduced. The incentive amounts will remain the same.

23.2. Reimbursement provided to subjects:[73]

N/A

23.3. Additional costs that subjects may be responsible for because of participation in the research:[74]

N/A

24. Setting

24.1. Site(s) or location(s) where the research team will conduct the research:

The clinics of the FQHCs involved in the research. Parents/legal guardians will be recruited in waiting rooms the day of their child's WCC visit and in between visits. When possible, private consultations rooms will be utilized to discuss the study with potential participants and obtain consent.

24.2. Composition and involvement of any community advisory board:

A Project Working Group (PWG) involving stakeholders representatives from the FQHCs community partners has been assembled. Please refer to IRB ID: STUDY00000413

24.3. For research conducted outside of the organization and its affiliates:[75]

24.3.1. Site-specific regulations or customs affecting the research:

N/A

24.3.2. Local scientific and ethical review structure:

N/A

25. Resources Available

25.1. Qualifications (e.g., training, education, experience, oversight) of investigator(s) to conduct and supervise the research:[76]

Principal Investigator: will be responsible for the overall design and management of the study. She will oversee the phase 2 RCT including the data collection efforts. The PI will work with the academic research team all aspects of the study. The PI is experienced in clinical research and in the area of well-child care clinical practice redesign.

Co-Investigators combined experience includes randomized clinical trials, studies of strategies to enhance the quality of care within primary care settings, study design, econometric methods, analysis of large administrative (claims) databases, and cost and cost-effectiveness analysis. Additionally, at least one Co-I has experience with community-based outreach workers, or patient navigators which are similar to parent coaches, electronic health record (EHR)-based innovations including incorporation of screening instruments and other components into the EHR workflow, and cost analyses of a variety of studies. Co-I responsibilities include meeting regularly with the PI, lending expertise on study design, fieldwork issues, and overall analytic strategy, dissemination, cost analysis, interpretation of cost data and development of logs used to track intervention staff time.

Other Personnel: The research staff will include a team who worked together on the pilot RCT of the intervention Parent-focused Redesign for Encounters, Newborns to Toddlers (PARENT). The team consists of the Project Manager, and three Research Assistants. The Project manager will manage all day-to- day needs of the project during all project years, and will continue to work closely with the PI. The research assistants will work with the project manager to supervise students and interns who will assist with data entry. The Research Assistants will collect survey data. However, the chart review data will be collected only by the Project Manager and trained Research Assistants.

25.2. Other resources available to conduct the research:[77]

None

26. Coordinating Center Procedures

26.1. Coordinating center institution:

There is no coordinating center for this study.

26.2. If Seattle Children's is the coordinating center:

26.2.1. Process to ensure communication among sites:[78]

The study PI, Dr. Coker, and the all research assistants and the project manager at SC and UCLA meet at least 3 times per week to discuss the study progression. The UCLA site PI, the study PI, and the project manager meet monthly.

26.2.2. Process to ensure all site investigators conduct the study according to the IRB approved protocol and report all non-compliance:

Dr. Coker (study PI) is in close contact with all the research study staff and meets with them as a team weekly to review enrolled participants, and any concerns or problems that they faced. She also is responsible for training and practicing mock enrollments using the approved protocol.

26.2.3. Process to ensure all required approvals are obtained at each site:

The PI and project manager work together to ensure that all approvals are obtained.

26.2.4. Process to ensure all sites are informed of any problems and/or interim results:

Since all staff in the study meet on a weekly basis, this is reviewed weekly. All staff are asked weekly to report any events or deviations from the protocol.

27. Good Clinical Practice

27.1. If you have committed to conducting the described study per International Center for Harmonization of Good Clinical Practice (ICH-GCP), check this box: ☐[79]

[1] Include information if this protocol is associated with other IRB-approved studies (e.g. is this application the next part/phase of a previously approved application.

[2] In clinical trials, an endpoint is an event or outcome that can be measured objectively to determine whether the intervention being studied is beneficial. Some examples of endpoints are survival, improvements in quality of life, relief of symptoms, and disappearance of the tumor.

[3] Include information on a drug or biologic in this section if: (1) the study specifies the use of an approved drug or biologic; (2) the study uses an unapproved drug or biologic; (3) the study uses a food or dietary supplement to diagnose, cure, treat, or mitigate a disease or condition; or (4) data regarding subjects will be submitted to or held for inspection by the Food and Drug Administration (FDA). Only include information on a device in this section if: (1) the study evaluates the safety or effectiveness of a device; (2) the study uses a humanitarian use device (HUD) for research purposes; or (3) data regarding subjects will be submitted to or held for inspection by the FDA. Please note that mobile medical applications may meet the definition of a device – see FDA Guidance.

[4] See the Investigator Manual HRP-103 for sponsor requirements for FDA-regulated research.

[5] Explain what IND exemption category applies to the drug and why. Note that a drug is not exempt from an IND unless all criteria for one category are met. See "HRP-306: Drugs" for more information.

[6] Explain what IDE exemption category applies to the device and why. Note that a device is not exempt from an IDE unless all criteria for one category are met. See "HRP-307: Devices" for more information.

[7] Explain why the device is NOT a significant risk device. A significant risk device means an investigational device that: (a) is intended as an implant and presents a potential for serious risk to the health, safety, or welfare of a subject; (b) is purported or represented to be for use supporting or sustaining human life and presents a potential for serious risk to the health, safety, or welfare of a subject; (c) is for a use of substantial importance in diagnosing, curing, mitigating, or treating disease, or otherwise preventing impairment of human health and presents a potential for serious risk to the health, safety, or welfare of a subject; or (d) otherwise presents a potential for serious risk to the health, safety, or welfare of a subject.

[8] Be sure to indicate if controls will be included and include information about why control arms are ethically acceptable.

[9] Describe all of the research procedures being performed. Be sure to make it clear which procedures apply to each subject population. When applicable, describe how research procedures differ from standard of care and/or affect standard of care. Describe any audio/video recording that will be involved.

[10] Attach all surveys, scripts, and data collection forms to the "Supporting Documents" page.

[11] Include information about the frequency of data collection.

[12] See HRP-001 - SOP – Definitions for definition of banking. Type N/A if not applicable. If the data is subject to NIH Genomic Data Sharing Policies (e.g. you will submit data to dbGaP, NDAR, FITBIR), indicate here.

[13] If applicable, include a list of identifiers that will be banked.

[14] Be general (e.g., researchers' lab, clinic, etc.)

[15] Generally, data and/or specimens should be released in a coded, non – identifiable manner.

[16] Include a description of the process used to verify and document that any required approvals have been obtained prior to release of data/specimens from the bank.

[17] You can allow for use for broad purposes

[18] This includes putting results and/or data in the subject medical records.

[19] If your population will differ from the representative population where the study will take place (e.g., race, ethnic group, or gender), provide a rationale for the differences.

[20] If you check a box below, be sure to include the additional safeguards associated with the population.

[21] Refer to HRP-416 CHECKLIST: Children.

[22] If the study is minimal risk, explain why. Must also include, as applicable: (1) why direct benefits are anticipated, (2) why risks are justified by anticipated benefit and/or the relationship between risk and prospective benefit compared to available alternatives, (3) why risk represents only minor increase over minimal risk, (4) how study procedures are reasonably commensurate with those inherent to the child's actual or expected conditions, (5) whether the interventions/procedures are likely to yield generalizable knowledge about the participant's condition and why it is of "vital importance" to understanding or amelioration of the participant's underlying disorder or condition, and (6) an explanation of what alternative methods/approaches were considered to make the above assessments (as applicable).

[23] This population may be wards of the state or any other agency, institution, or entity. Refer to HRP-416 CHECKLIST: Children, Section 6, for additional guidance on required considerations for this population.

[24] This refers to both cognitive impairments and adults who are incapacitated for any other reason. As applicable, refer to HRP-417 CHECKLIST: Cognitively Impaired Adults.

[25] Refer to HRP-413 CHECKLIST: Neonates and HRP-414 CHECKLIST: Neonates of Uncertain Viability.

[26] Refer to HRP-412 CHECKLIST: Pregnant Women.

[27] Refer to HRP-415 CHECKLIST: Prisoners

[28] Indicate how you will ensure that there is no coercion or undue influence

[29] A subject is considered "enrolled" when they consent to be in the study.

[30] Only applicable for multisite studies.

[31] i.e., numbers of subjects excluding screen failures.

[32] Payment for participation is not considered a benefit.

[33] For example, data will be double entered, data will be reviewed by another study team member to ensure accuracy, etc.

[34] If your study is multisite and there are differences in how confidentiality will be maintained by the coordination center and our local site, this should be explained in this section (e.g. local site will have samples that are linked to a person's name, but the coordination center will only receive coded samples without any links). Confidentiality regarding use of Social Media will be explained in a protocol section below.

[35] Applicable for studies that present more than minimal risk.

[36] Include information about who (describe in terms of role or group) will review the data.

[37] This should be specific to the social media you are using for the research.

[38] All communications that are directed towards subjects and specific to a particular study will require prior IRB review and approval. All non-IRB reviewable communications can be described in general terms by category – news stories, relevant publications – and representative examples of each can be provided.

[39] Applicable if the research involves more than minimal risk to subjects. If minimal risk, this section is N/A.

[40] If this is a multicenter study and subjects will be recruited by methods not under the control of the local site (e.g., call centers, national advertisements) those methods should also be described here.
[41] "Privacy interest" refers to a person's desire to place limits on whom they interact or whom they provide personal information.
[42] For example: medical records, CIS, clinical databases, other study records. If the study will access PHI for recruitment purposes without prior authorization from subjects, please address this in the HIPAA Authorization section below.
[43] Attach copies of these documents to the Recruitment Materials section of the study SmartForm. For printed advertisements, attach the final copy. For online advertisements, attach the final screen shots (including any images). When advertisements are taped for broadcast, send the final audio/video tape to IRB@seattlechildrens.org. You may attach the wording of the advertisement to the SmartForm prior to taping to preclude re-taping because of inappropriate wording, provided the IRB reviews the final audio/video tape.
[44] "Privacy interest" refers to a person's desire to place limits on whom they interact or whom they provide personal information.
[45] This section describes the way(s) in which the processes for this study will not follow Seattle Children's SOP.
[46] See HRP-090, HRP-091, and Investigator Manual HRP-103 for more information.
[47] Note the Short Form Consent may only be used when certain conditions are met. See HRP-091 for requirements for Short Form consent form use.
[48] Seattle Children's IRB prohibits the exclusion of non-English speaking populations from research unless there is sufficient justification for the exclusion. See Investigator Manual HRP-103 for more information.
[49] For research conducted in the state, review "SOP: Legally Authorized Representatives, Children, and Guardians (HRP-013)" to be aware of which individuals in the state meet the definition of "children." The age of majority in Washington is 18; however, sometimes younger children have ability to consent for certain types of care (e.g. sexual reproduction/health; mental health; drug/alcohol treatment). For research conducted outside of the state, provide information that describes which persons have not attained the legal age for consent to treatments or procedures involved the research, under the applicable law of the jurisdiction in which research will be conducted. One method of obtaining this information is to have a legal counsel or authority review your protocol along the definition of "children" in "SOP: Legally Authorized Representatives, Children, and Guardians (HRP-013)." If the sites in other states in the study are conducting their own IRB review, you do not need to worry about this--type N/A. If you are conducting research and are actively recruiting participants outside of Washington who are NOT coming to SCH to give consent and who will be covered under SCH IRB approval, this section should be addressed in your protocol.
[50] For minimal risk studies and greater than minimal risk studies that offer a prospect of benefit, the IRB generally requires one parent to provide permission for the child to participate.
[51] If parental permission will not be obtained, please address this in the Waiver or Alteration of Consent Process below.
[52] See HRP-013 for more information.
[53] The IRB generally follows the following guidelines for written assent: children 7-12 should provide written assent on the "simple" assent form (HRP-502G); children 13-17 should provide written assent by co-signing the parental permission form (HRP-502A). The IRB will consider other assent scenarios (e.g. verbal assent for some or all children; not requiring assent for some or all children; or waiving assent): please provide details about the plan for your study. See HRP-090 and HRP-416 for more information on waiving assent and when assent is not necessary.
[54] See Appendix A-13 of the Investigator Manual HRP-103 for requirements for re-consent at age 18. If you think you meet the conditions for a waiver at 18, please address this in the Waiver or Alteration of Consent Process below.
[55] See "HRP-417 Cognitively Impaired Adults" for further information.
[56] For example: durable power of attorney for health care, court appointed guardian for health care decisions, spouse, and adult child. If you are following HRP-013 in order to make this determination, simply state that in this section. For research conducted in the state, review "SOP: Legally Authorized Representatives, Children, and Guardians (HRP-013)" to be aware of which individuals in the state meet the definition of "legally authorized representative." For research conducted outside of the state, provide information that describes which individuals are authorized under applicable law to consent on behalf of a prospective subject to their participation in the procedure(s) involved in this research. One method of obtaining this information is to have a legal counsel or authority review your protocol along the definition of "legally authorized representative" in "SOP: Legally Authorized Representatives, Children, and Guardians (HRP-013)." If the sites in other states in the study are conducting their own IRB review, you do not need to worry about this--type N/A. If you are conducting research and are actively recruiting participants outside of Washington who are NOT coming to Washington to give consent and who will be covered under SCH IRB approval, this section should be addressed in your protocol.
[57] The IRB may allow the person obtaining assent to document assent on the consent document.
[58] For example: consent/parental permission will not be obtained, required information will not be disclosed, the research involves deception, waiver for participants who turn 18, waiver for information collected about a non-present parent, or other waivers as necessary.
[59] The IRB needs to make all the waiver findings and key to this determination is that the IRB understand why it is not practicable to do the research without a waiver of consent. You need to provide a rationale in order for the IRB to consider whether the waiver criteria are met. See "HRP-410: Waiver or Alteration of the Consent Process" for further information.
[60] Possible reasons might include: a) you are not collecting information that could put subjects or their families at harm, e.g., affect eligibility for insurance, employability, stigmatization; b) you are not collecting information that would alter or affect the subject's care; c) any publication or presentation of research results would be done in a manner that would never reveal an individual's identity either directly or indirectly.
[61] Possible reasons could be: a) inability to locate families because of the lengthy time period over which the records/samples were created; b) many of the subjects whose records, data, or specimens will be used may have died and contacting the families about the research could cause harm and anguish to families; c) all eligible patients must be included in the study for the results to be meaningful.
[62] See "HRP 419: Waiver of Consent for Emergency Research" for further information.
[63] This section describes the ways in which the procedures will not be following Seattle Children's SOP.
[64] See "HRP-411: Waiver or Written Documentation of Informed Consent" for further information.
[65] An information sheet template can be found in the Click IRB Library and should be attached to the consent form of the study SmartForm. For internet research, the information sheet can be translated to an on-line format, if desired.
[66] The IRB sometimes requires a script if you are having the consent conversation over the phone rather than in person. Templates for a consent script are available on the IRB website on the Participant Recruitment page and should be attached to the study SmartForm.
[67] PHI is health information that is also identifiable because it includes one or more of the 18 HIPAA identifiers. See Investigator Manual HRP-103 for the list of HIPAA identifiers.
[68] If your study involves using or creating PHI and your only contact with participants is online, you can request an alteration of HIPAA authorization to remove the signature requirement. As an alternative to a waiver of documentation of consent and an alteration of HIPAA authorization, you must demonstrate that the electronic consent signatures are compliant with applicable state/international law (in Washington, see RCW 19.34.300).
[69] For example: altering HIPAA elements for international research.
[70] Possible reason could be: the nature of the research is specific to individuals' health and requires access to individuals' health records.
[71] See "HRP-316: Payments" for further information.
[72] Methods of payment include check, ClinCard, gift cards, etc. Provide details on who will be the recipient of the payment (parent or child).

[73] Reimbursement is used when the subject is paid back for travel expenses such as transportation, food, childcare, or lodging. Reimbursement is generally distributed to person who incurred cost (usually parent) and requires receipts to be submitted.

[74] This could include things like fuel/transportation costs, parking, and/or childcare.

[75] Type N/A if this section does not apply.

[76] Provide enough information to convince the IRB that the principal and/or co-investigator(s) are appropriately qualified to conduct and supervise the proposed research. When applicable, describe their prior clinical experience with the test article or study-related procedures, or describe their knowledge of the local study sites, culture, and society.

[77] For example, as appropriate: (1) Justify the feasibility of recruiting the required number of suitable subjects within the agreed recruitment period. For example, how many potential subjects do you have access to? What percentage of those potential subjects do you need to recruit? (2) Describe the time that you will devote to conducting and completing the research. (3) Describe the facilities in which the research will be conducted. (4) Describe the availability of medical or psychological resources that subjects might need as a result of an anticipated consequences of the human research. (5) Describe your process to ensure that all persons assisting with the research are adequately informed about the protocol, the research procedures, and their duties and functions.

[78] Including communication between sites of current study document versions and modifications.

[79] See your contract/agreement or Sponsor Documentation if you are unsure
