# Supplementary material for: Cost Analysis of the PARENT Trial of Community Health Workers in Early Childhood Preventive Care: A Secondary Analysis of a Cluster-Randomized Clinical Trial
Source: JAMA Netw Open. 2025 Jul 31;8(7):e2522732. doi: 10.1001/jamanetworkopen.2025.22732 (PMC12314717; doi:10.1001/jamanetworkopen.2025.22732)
Supplement: Supplement 3. — Data Sharing Statement [file jamanetwopen-e2522732-s003.pdf]

Data Sharing Statement

Coker. Cost Analysis of the PARENT Trial of Community Health Workers in Early Childhood Preventive Care. *JAMA Netw Open*. Published July 31, 2025.
doi:10.1001/jamanetworkopen.2025.22732

Data
Additional Information: NCT03797898
Data available: Yes
Data types: Deidentified participant data
How to access data: tumaini.coker@seattlechildrens.org
When available: With publication

Supporting Documents
Document types: None

Additional Information
Who can access the data: researchers whose proposed use of the data has been approved
Types of analyses: for secondary data analyses for research
Mechanisms of data availability: after approval of a proposal, with investigator support
